# mRNA localization is linked to translation regulation in the *Caenorhabditis elegans* germ lineage

**DOI:** 10.1242/dev.186817

**Published:** 2020-07-08

**Authors:** Dylan M. Parker, Lindsay P. Winkenbach, Sam Boyson, Matthew N. Saxton, Camryn Daidone, Zainab A. Al-Mazaydeh, Marc T. Nishimura, Florian Mueller, Erin Osborne Nishimura

**Affiliations:** 1 Department of Biochemistry and Molecular Biology, Colorado State University, Fort Collins, CO 80523, USA; 2 Department of Biology and Biotechnology, Hashemite University, Zarqa, 13115, Jordan; 3 Department of Biology, Colorado State University, Fort Collins, CO 80523, USA; 4 Département Biologie Cellulaire et Infections, Unité Imagerie et Modélisation, Institut Pasteur and CNRS UMR 3691, 28 rue du Docteur Roux, 75015 Paris, France

**Keywords:** mRNA localization, *C. elegans*, P granule

## Abstract

*Caenorhabditis elegans* early embryos generate cell-specific transcriptomes despite lacking active transcription, thereby presenting an opportunity to study mechanisms of post-transcriptional regulatory control. We observed that some cell-specific mRNAs accumulate non-homogenously within cells, localizing to membranes, P granules (associated with progenitor germ cells in the P lineage) and P-bodies (associated with RNA processing). The subcellular distribution of transcripts differed in their dependence on 3′UTRs and RNA binding proteins, suggesting diverse regulatory mechanisms. Notably, we found strong but imperfect correlations between low translational status and P granule localization within the progenitor germ lineage. By uncoupling translation from mRNA localization, we untangled a long-standing question: Are mRNAs directed to P granules to be translationally repressed, or do they accumulate there as a consequence of this repression? We found that translational repression preceded P granule localization and could occur independently of it. Further, disruption of translation was sufficient to send homogenously distributed mRNAs to P granules. These results implicate transcriptional repression as a means to deliver essential maternal transcripts to the progenitor germ lineage for later translation.

## Abstract (precis)

**Summary:** Maternally loaded mRNAs localize non-homogenously within *Caenorhabditis elegans* early embryos, correlating with their translational status and lineage-specific fates

## INTRODUCTION

The progression of life from two gametes to an embryo involves the transfer of gene expression responsibilities from the parental to zygotic genomes. In animals, this maternal-to-zygotic transition requires a pause in transcription during late oogenesis, fertilization and the first stages of zygotic development (Hamm and Harrison, 2018; Robertson and Lin, 2015; Schulz and Harrison, 2019; Vastenhouw et al., 2019). Until zygotic transcription resumes, cell-type transcriptome differences in the early embryo arise through post-transcriptional mechanisms acting on mRNAs inherited from the parental gametes.

In *Caenorhabditis elegans*, transcriptional repression initiates in late oogenesis by an unknown mechanism (Gibert et al., 1984; Walker et al., 2007), but is sustained in post-fertilization stages by sequestration of transcriptional machinery to the cytoplasm (Guven-Ozkan et al., 2010). Transcription resumes 2 h postfertilization, initiating in the somatic cells of four-cell embryos and culminating in the P_4_ cell of the primordial germ lineage (P lineage) at the 28-cell stage (Seydoux and Fire, 1994; Seydoux et al., 1996).

Even in the absence of *de novo* zygotic transcription, the transcriptomes of early *C. elegans* blastomeres diversify. Single cell resolution RNA-seq (scRNA-seq) assays have determined that the first two daughter cells (AB and P_1_) contain 80 AB-enriched and 201 P_1_-enriched transcripts distinguishing them (Osborne Nishimura et al., 2015). Similar approaches have identified additional maternally inherited transcripts with biased representation in different lineages through the first four cell divisions (Tintori et al., 2016). These cell-specific transcripts likely arise through post-transcriptional mechanisms of mRNA decay, mRNA stabilization or by movement (active or passive) of transcripts into distinct regions of dividing cells.

Interestingly, there is no reason *a priori* for transcriptome diversification to be required for cell-specific protein production. Translational control plays a major role in driving protein production during germline development (Merritt et al., 2008) and into early embryogenesis. Indeed, a major class of mutants that affect early cell fate development are cell-specific RNA binding proteins (RBPs), the target transcripts of which are translated with spatiotemporal specificity (D'Agostino et al., 2006; Jadhav et al., 2008; Oldenbroek et al., 2012, 2013).

Still, the mRNA encoding Negative Effect on Gut development (NEG-1; a cell fate determinant) has an anterior bias preceding anterior NEG-1 protein production, suggesting that patterns in mRNA localization can precede or even be amplified at the translation step (Elewa et al., 2015; Osborne Nishimura et al., 2015). Therefore, maternal asymmetric mRNAs appear to be important for cellular diversification in early development. In this study, we explore the mechanisms and functions of this patterning.

We report that several maternally inherited transcripts localize to subcellular regions within individual cells. In general, the anterior-biased (AB cell-enriched) transcripts tended to localize to cell-peripheral regions, often where the proteins they encode function. In contrast, posterior-biased (P_1_ cell-enriched) transcripts formed clustered granules overlapping with P granules, membraneless compartments of RNAs and proteins that form liquid-liquid phase separated condensates or hydrogels that mark the progenitor germ lineage (Seydoux, 2018; Marnik and Updike, 2019).

Understanding the functional roles of P granules (and other phase-separated condensates) is a current major challenge. In early embryos, P granules are dispersed in the cytoplasm and highly dynamic (Hird et al., 1996; Strome and Wood, 1982), but later grow into larger granules that coalesce around the nucleus (Sheth et al., 2010). Here, they extend the nuclear pore complex environment and branch into more specialized condensates such as mutator foci (Phillips et al., 2012) and Z-granules (Wan et al., 2018). Worms can recover from P granule disruption in early embryonic stages to properly specify the germline (Gallo et al., 2010), but later or sustained dysregulation leads to perturbations in germ-cell development (Wang et al., 2014), disruption of gene expression regulatory control (Campbell and Updike, 2015; Updike et al., 2014; Voronina et al., 2012) and fertility defects (Kawasaki et al., 2004; Spike et al., 2014; Wang et al., 2014). The reasons why mRNAs associate with P granules may depend on the individual transcript or developmental stage, but functions such as translational repression, RNA processing, small RNA-based regulation or piRNA licensing are possibilities, based on the functions of the proteins that compose P granules.

Here, we identify several new mRNA transcripts associated with P granules and observe that many are lowly translated. Indeed, the well-studied P granule-resident mRNA *nos-2* is also translationally repressed at early embryonic stages. Later, this repression is relieved when NOS-2 becomes essential for germline development (D'Agostino et al., 2006; Jadhav et al., 2008; Subramaniam and Seydoux, 1999). It is possible that mRNA transcripts, such as *nos-2* and others*,* associate with P granules to promote translational repression. Alternatively, transcripts may accumulate in P granules after repression as a downstream step. In this study, we find that translational repression of *nos-2* mRNA precedes *nos-2* mRNA accumulation in P granules and can persist without P granule localization, supporting the second model. Further, we found that loss of translation can direct homogenously distributed transcripts to P granules, again suggesting that localization is a downstream step.

Overall, our work expands the list of membrane-associated mRNAs (from 0 to 5) and P granule-associated mRNAs (from roughly 10 to 16). Our findings also suggest that the subcellular patterning of maternally inherited transcripts is a common feature of early embryogenesis. By identifying and studying additional mRNAs with subcellular localization in the *C. elegans* early embryo, we can better determine mechanisms and purposes of their localization in early development.

## RESULTS

### Maternally inherited mRNA transcripts display subcellular localization

**Fig. 1.**
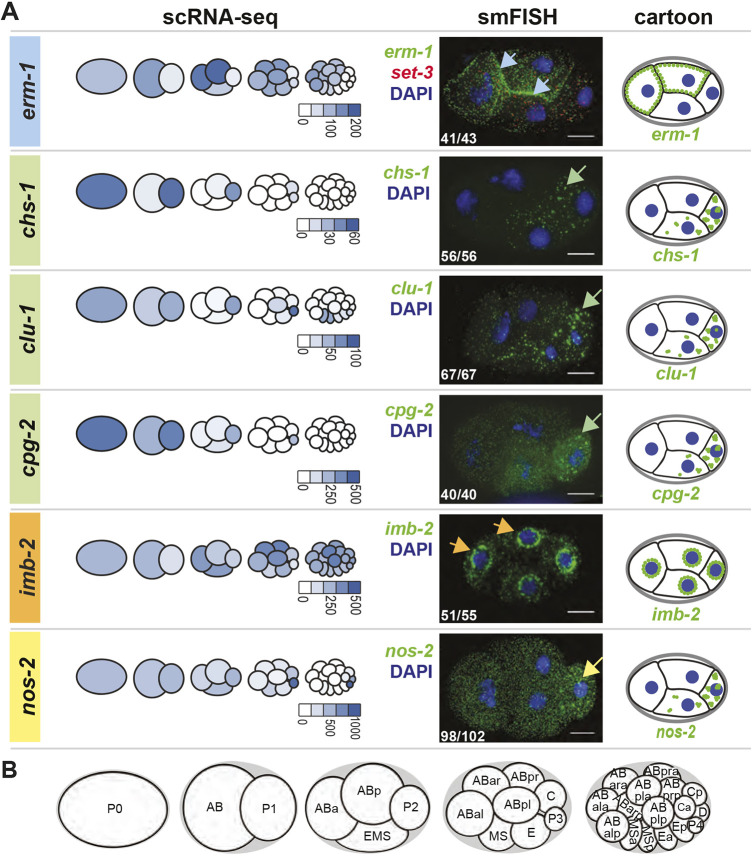
**Subcellular localization patterns of maternally inherited mRNAs.** (A) mRNA localization patterns for *erm-1*, *chs-1*, *clu-1*, *cpg-2*, *imb-2* and *nos-2* are shown (Table 1, Fig. S1A). They represent AB-enriched (blue), P_1_-enriched (green) and symmetric (orange) maternal mRNA and a known P granule control (yellow). Left column shows the pattern of mRNA abundance through the first four cell divisions as previously reported using scRNA-seq data (Tintori et al., 2016), illustrated as a proportionally colorized pictograph. Normalized transcript abundance values are indicated below each pictograph. Center column shows mRNA imaging using smFISH of a representative four-cell embryo, showing the mRNA of interest (green), DNA (DAPI; blue), and *set-3* [SET (trithorax/polycomb) domain containing; red] as a symmetric control. *set-3* was co-probed in each embryo but only shown once for simplicity. mRNAs were found concentrated at cell peripheries (*erm-1*, blue arrows), into clusters (*chs-1*, *clu-1* and *cpg-2*, green arrows), at nuclear peripheries (*imb-2*, orange arrows) or at known P granules (*nos-2*, yellow arrow). Inset white numbers represent the number of times the pattern was observed out of the total four-cell-stage embryos surveyed over a minimum of five biologically replicated experiments. Right column shows cartoon depictions of each mRNA of interest (green), shown to summarize subcellular distribution patterns. (B) Cartoon depictions of the first five embryonic stages. Scale bars: 10 µm.

scRNA-seq assays have identified transcripts that are differentially abundant between cells before the onset of zygotic transcription in *C. elegans* (Hashimshony et al., 2012, 2015; Osborne Nishimura et al., 2015; Tintori et al., 2016). To verify the cell-specificity of these mRNAs and visualize their localization, we selected several to image in fixed *C. elegans* embryos using single-molecule resolution imaging [single-molecule fluorescence *in situ* hybridization (smFISH) or single-molecule inexpensive fluorescence *in situ* hybridization (smiFISH)]. We chose eight AB-enriched transcripts, eight P_1_-enriched transcripts, four uniformly distributed (maternal) transcripts and eight zygotically expressed transcripts. Single-molecule resolution imaging confirmed the cell-specific patterning predicted by RNA-seq for seven out of eight AB-enriched, seven out of eight P_1_-enriched transcripts, and four out of four symmetric transcripts. Strikingly, many maternally inherited transcripts yielded subcellular localization patterns beyond cell-specific patterning (Table 1, Fig. 1, Movies 1-6, Fig. S1).

**Table 1.**
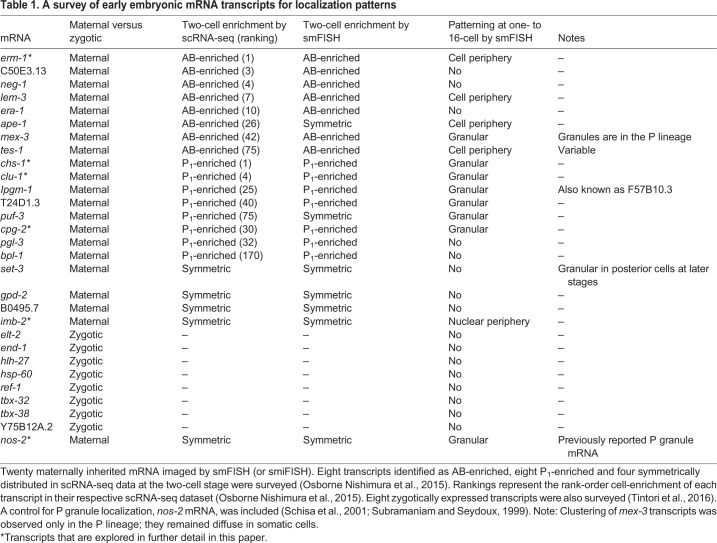
**A survey of early embryonic mRNA transcripts for localization patterns**

AB-enriched transcripts tended to localize to cell peripheries (Table 1). Specifically, AB-enriched *erm-1* (*Ezrin/Radixin/Moesin*), *lem-3* (*LEM domain protein*), *ape-1* (*APoptosis Enhancer*) and *tes-1* (*TEStin homolog*) mRNAs accumulated there. ERM-1 protein also accumulates at cell-to-cell contacts where it functions in the remodeling of apical junctions (Van Fürden et al., 2004). Similarly, LEM-3, a nucleic acid metabolizing enzyme, localizes to cell membranes (supplemental material of Dittrich et al., 2012) and cytoplasmic foci. The localization of APE-1 and TES-1 proteins are uncharacterized, but they contain domains known to associate with membranes (ankyrin-repeat domain in APE-1 and PET domain in TES-1) (Bennett and Baines, 2001; Sweede et al., 2008). For this paper, we focused on *erm-1* as a representative of this group (Fig. 1).

P_1_-enriched transcripts primarily aggregated in RNA granules in the P lineage (Table 1, Fig. 1, Fig. S1). This included transcripts important in eggshell formation such as *chs-1* (*CHitin Synthase*) and *cpg-2* (*Chondroitin ProteoGlycan*), mitochondrial distribution and stress response such as *clu-1* [*yeast CLU-1 (CLUstered mitochondria) related*], as well as the carbohydrate-metabolizing enzyme F57B10.3 (recently renamed *ipgm-1*; *cofactor-Independent PhosphoGlycerate Mutase homolog*) (Fields et al., 1998; Maruyama et al., 2007; Olson et al., 2012; Zhang et al., 2004).

Of the maternally inherited transcripts that distribute symmetrically at the two-cell stage, only one of four tested showed subcellular patterning (Table 1, Fig. S1). The transcript *imb-2* (*IMportin Beta family*) localized to nuclear peripheries, coincident with its encoded protein, an Importin-β homolog that facilitates nuclear pore complex import (Fig. 1). In no cases did we observe subcellular localization for mRNAs expressed zygotically, suggesting that subcellular patterning is more common among maternally inherited transcripts that those zygotically transcribed. However, because zygotically dividing cells subdivide successively, beyond the 16-cell stage their reduced size could potentially obscure our ability to call their localization accurately (Table 1).

**Fig. 2.**
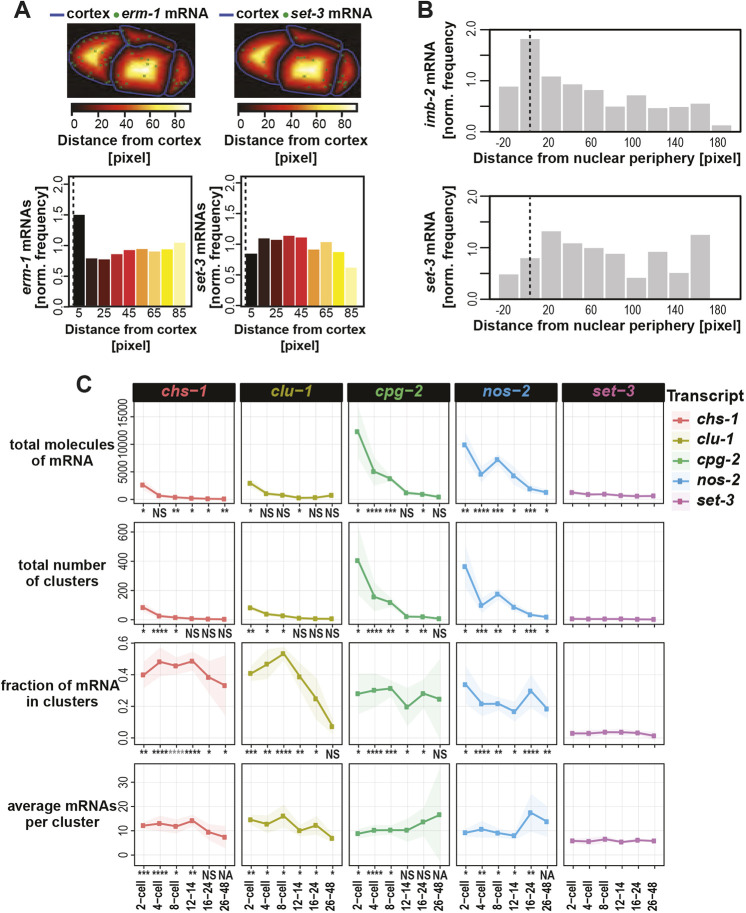
**Quantification of mRNA and their patterning.** (A) The number of mRNA molecules (green dots) located within binned distances from the cell cortex (blue lines) were tabulated and normalized against the total volume of each concentric space. The frequencies with which *erm-1* mRNA and *set-3* mRNA occurred at varying distances in one embryo are shown. (B) The frequencies with which mRNA appeared in relation to the nuclear peripheries in one embryo were similarly calculated for *imb-2* mRNA and *set-3* mRNA. (C) Several metrics of clustering were quantified for: *chs-1* (red), *clu-1* (ochre), *cpg-2* (green), the P granule mRNA of *nos-2* (blue) and for comparison *set-3* (purple)*.* We calculated the total number of RNAs in each embryo, the total number of clusters identified in each embryo, the fraction of total mRNAs located within clusters, and the average estimated number of mRNA molecules per cluster within a given embryo. The average of each metric and their standard deviation (shading) for each transcript at six cell stages are shown, representing a minimum of five embryos for each type and time point over a minimum of three replicates. Significance indicates *P*-values derived from multiple test corrected *t*-tests comparing the transcript of interest versus the control transcript *set-3* for the metric of interest at the given stage. Adjusted *P* value legend: NS>0.05; 0.05>*>0.005; 0.005>**>0.0005; 0.0005>***>0.00005; 0.00005>****.

In addition to these surveyed transcripts, we also used smFISH to image *nos-2*, a previously reported mRNA resident of P granules required for germline maintenance and fertility (Subramaniam and Seydoux, 1999) (Table 1, Fig. 1). smFISH verified P granule localization of *nos-2* mRNA and showed that granular patterning was coincident with P lineage enrichment – both beginning at late four-cell stage (Fig. 2C, Fig. S2).

To explore the dynamics of subcellular patterning through embryogenesis, we imaged key transcripts from the one-cell stage through hatching. The onset and persistence of subcellular mRNA localization varied depending on the transcript and its biology (Fig. S2). *chs-1* mRNA first localized to posterior clusters at the one- or two-cell stage but degraded over successive cell divisions until dissipating by the 48-cell stage (Fig. S2), whereas *imb-2* appeared at or near nuclear membranes in all stages assayed. This is consistent with the roles of the proteins as CHS-1 is essential primarily for deposition of chitin in the eggshell between oogenesis and egg-laying (Zhang et al., 2005), whereas the IMB-2 protein is required throughout the life of the worm for nuclear import (Putker et al., 2013). In contrast to *chs-1*, *nos-2* mRNA distributed homogenously before the four-cell stage and then began clustering in the P lineage, coincident with its degradation in somatic cells. *nos-2* mRNA clusters grew in size until the 28-cell stage (Fig. S2). At the 28-cell stage, *nos-2* transcripts became visible as individuals in the cytoplasm, concurrent with a decrease in the size of *nos-2* mRNA clusters. Translational regulation of *nos-2* is dynamic during these stages. *nos-2* mRNA is translationally repressed before the 28-cell stage, at which point translation repression is relieved (D'Agostino et al., 2006; Jadhav et al., 2008). Therefore, the transition in RNA localization accompanies this transition in regulatory status. What was more surprising is that *nos-2* mRNA could both be observed as individual mRNAs and localized into granules before the 28-cell stage during its phase of translational repression. During the one-, two- and early 4-cell stages, *nos-2* mRNA fails to produce protein, but also does not localize to clusters, illustrating that these processes can be uncoupled. Altogether, subcellular transcript localization appears transient or persistent depending on the encoded function of the mRNA.

### Quantification strategies to characterize mRNA patterning

To better describe the subcellular mRNA patterns we observed, we detected individual mRNA molecules in 3D images using FISH-quant (Mueller et al., 2013) and developed metrics to describe their localizations at membranes or within clusters.

*erm-1* mRNA localized to cell peripheries. To characterize this propensity in an unbiased manner, we calculated the frequency with which *erm-1* transcripts accumulated at increasing distances from cell membranes (Fig. 2A). After normalizing for the decreasing volumes of each concentric space, we determined that *erm-1* mRNA were twice as likely to occur within 5 μm of a cell membrane than more than 5 μm from one. In contrast, homogenously distributed *set-3* (*SET domain containing*) transcripts were equally likely to be present at all distances (both measured using 10 μm bin sizes) (Fig. 2A).

Similarly, we calculated the frequency of *imb-2* mRNA at increasing distances from the nuclear periphery (Fig. 2B). *imb-2* transcripts were twice as abundant within 10 μm from the nuclear membrane than at 10 μm or more from a nuclear membrane, again adjusting for volumes of these spaces. The more ubiquitous *set-3* transcripts showed no nuclear peripheral-enrichment.

In developing metrics of mRNA clustering, we found that overlapping mRNA signals complicated the ‘single molecule’ nature of smFISH, which relies on sufficient spacing between individual transcripts. To overcome this, we used a tiered approach, first identifying individual mRNAs (Mueller et al., 2013) before estimating the number of molecules contributing to signal overlap by fitting a Gaussian mixture model (GMM) to the average fluorescence intensities and volumes of the individual molecules (see Materials and Methods). Deconvolved mRNA molecules could then be separated into clusters using a geometric nearest neighbor approach (Ester et al., 1996).

To characterize mRNA clusters, we quantified total number of mRNA molecules per embryo, total number of mRNA clusters per embryo, fraction of total mRNAs that localize into clusters (as opposed to individuals), and estimated number of mRNAs within each cluster. We calculated these measurements for four clustered transcripts (*chs-1*, *clu-1*, *cpg-2* and *nos-2*) at six stages of embryonic development (Fig. 2C). *cpg-2* and *nos-2* were the most abundant transcripts (∼10,000 molecules per embryo) in contrast to *chs-1* or *clu-1* (∼2500 molecules per embryo) at the same time point (two-cell stage). The number of *cpg-2* and *nos-2* mRNA molecules comprising each cluster increased over time, whereas *chs-1* and *clu-1* did not. For *nos-2*, mRNA accumulated to a maximum of 20 molecules per cluster at the 24-cell stage, just before *nos-2* translational activation. After this point, *nos-2* mRNA clusters decreased in size, appearing dispersed in the cytoplasm. All clustered transcripts exhibited marked differences in clustering statistics from the homogenously distributed *set-3* transcripts.

### Clustered transcripts co-localize with markers of P granules and, less frequently, with markers of P-bodies

**Fig. 3.**
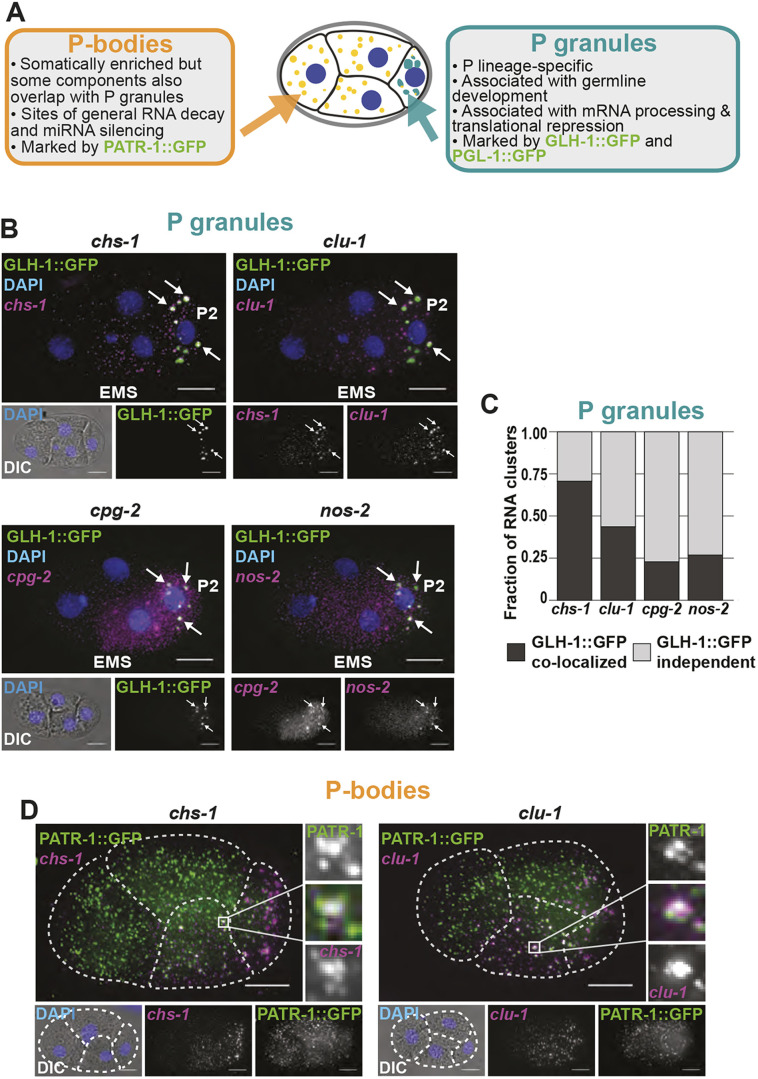
**Posterior clustered mRNAs co-localize with P granules and P-bodies.** (A) Schematic detailing how P granules are distinct from P-bodies. (B) Fixed embryos were imaged for the P granule marker GLH-1::GFP (green) and *chs-1*, *clu-1*, *cpg-2* or *nos-2* transcripts (magenta). DNA (DAPI, blue) and differential interference contrast microscopy (DIC) are also shown. (C) The fraction of mRNA clusters overlapping with P granules (dark gray) and P granule-independent clusters (light gray) in four-cell embryos was calculated by assessing spatial overlap between mRNA clusters and GLH-1::GFP-marked P granules. (D) Fixed embryos were imaged for the P-body protein marker PATR-1::GFP amplified using immunofluorescence (green) with smFISH imaging of *chs-1* mRNA or *clu-1* mRNA (magenta), and DNA (DAPI; blue). Enlargements of boxed areas illustrate regions of co-localization. Dashed white lines indicate cell boundaries.

mRNA clustering is typically indicative of localization into granules. Many types of condensates exist, such as stress granules (associated with translationally repressed transcripts that accumulate during stress), P-bodies (processing bodies, associated with RNA processing enzymes) and germ granules (associated with regulatory control in animal germ cells). In *C. elegans*, germ granules are specifically called P granules in the early embryo (Fig. 3A) (Seydoux, 2018; Marnik and Updike, 2019) and they segregate to the P lineage with each successive cell division. Dual mechanisms of preferential coalescence/segregation in the P lineage and disassembly/degradation in somatic cells drives their concentration in the P lineage (Brangwynne et al., 2009; DeRenzo et al., 2003; Wang et al., 2014).

Given that we observed *chs-1*, *clu-1* and *cpg-2* mRNAs clustered and progressing down the P lineage, we hypothesized that they might be within P granules. To test this, we imaged *chs-1*, *clu-1*, *cpg-2* and, for comparison, *nos-2* by smFISH in worms expressing P granule markers GLH-1::GFP (Fig. 3B) or PGL-1::GFP (Fig. S3). mRNA clusters overlapped with both P granule markers. Indeed, 23% (*cpg-2*) to 75% (*chs-1*) of identified mRNA clusters overlapped with GLH-1::GFP-marked P granules at the four-cell stage (Fig. 3C), and their co-occurrence increased thereafter. Larger mRNA clusters were more likely to co-occupy space with P granules (Fig. S4). Conversely, 13-57% of GLH-1::GFP marked P granules contained an mRNA cluster of any specific transcript, suggesting some heterogeneity in their content. Together, these findings illustrate that P-lineage-enriched mRNA clusters in this study are P granule-associated RNAs.

Depending on the transcript, 25-75% of RNA clusters were distinct from P granule markers at the four-cell stage. These occurred in P cells and their sisters (most evidently in the EMS cell). Because many of the clustered mRNAs (*chs-1*, *clu-1*, *cpg-2* and *nos-*2) degrade in early embryogenesis (Fig. 2C), we hypothesized that the RNA clusters that did not overlap with P granule markers were P-bodies. P-bodies – as opposed to P granules – are associated with RNA decay as they contain high concentrations of RNA degrading proteins (DCAP-1, Argonaute, and Xrn-1) (Parker and Sheth, 2007) (Fig. 3A). In *C. elegans*, P granules and P-bodies share some protein components, but specific proteins distinguish each (Gallo et al., 2008; Voronina et al., 2011). To test our hypothesis, we imaged *chs-1*, *clu-1*, *cpg-2* and *nos-2* using smFISH concurrently with PATR-1::GFP (yeast PAT-1 Related) amplified by immunofluorescence to mark P-bodies (Materials and Methods, Fig. S5). *chs-1* and *clu-1* transcripts were enriched in posterior cells whereas PATR-1::GFP predominantly localized to somatic cells. However, within their regions of overlap, we identified co-localized clusters, indicating that some clusters of *chs-1* and *clu-1* mRNAs reside within P-bodies (Fig. 3D). Some *chs-1* and *clu-1* mRNA clusters failed to overlap with P granule or P-body markers, leaving their identity unknown. Whether these mRNA clusters are stable or short-lived is currently unclear, as fixed smFISH assays cannot resolve their dynamics.

Curiously, we noticed that transcripts did not mix homogenously within P granules but occupied discrete regions within granules. For example, *clu-1* mRNA typically surrounded a *chs-1* mRNA core (Fig. S6). These observations are echoed by other reports of homotypic mRNA spatial separation within germ granules (Eagle et al., 2018; Trcek et al., 2015) and suggest a complex organization to granules and the mRNAs they contain.

### 3′UTRs were sufficient to direct mRNAs to P granules but not membranes

The 3′ untranslated regions (UTRs) of transcripts have been implicated in driving subcellular localization of mRNAs in many organisms (Martin and Ephrussi, 2009). To determine whether 3′UTRs of transcripts in our study were sufficient to direct mRNA localization, we appended 3′UTRs of interest onto *mNeonGreen* reporters expressed from the *mex-5* promoter in transgenic strains. We generated single-copy chromosomal integrations using Cas9-mediated insertion into MosSci integration sites. We imaged *mNeonGreen* mRNA localization using *mNeonGreen* smFISH probes alongside probe sets for endogenous mRNA in the same embryos.

**Fig. 4.**
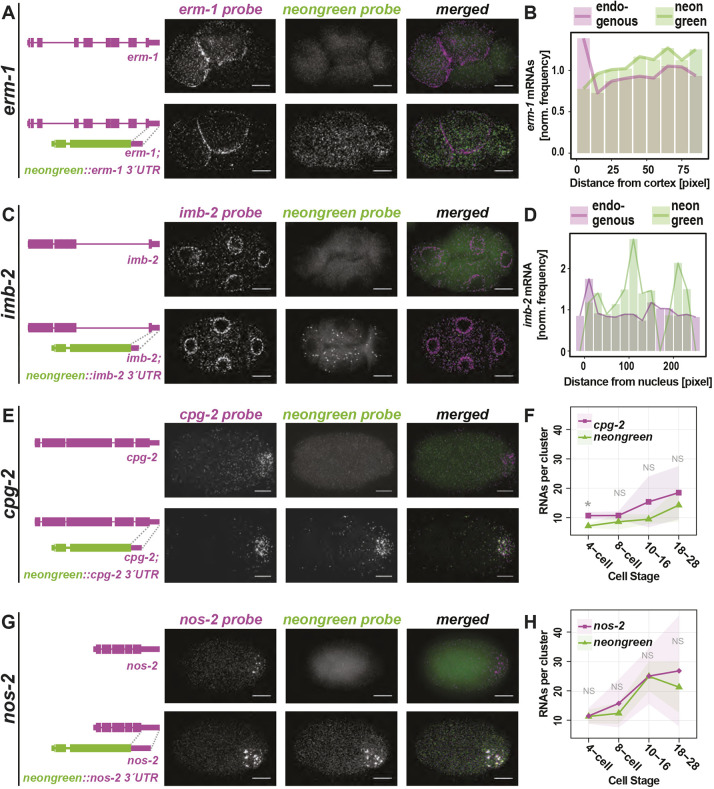
**3′UTRs of clustered, but not membrane-associated, transcripts are sufficient for subcellular localization.** (A,C,E,G) The 3′UTRs of *erm-1* (A), *imb-2* (C), *cpg-2* (E) and *nos-2* (G) were appended to monomeric NeonGreen (*mex-5p::mNeonGreen::3′UTR of interest*) and transgenically introduced as a single copy insert into otherwise wild-type worms. Wild-type control strains (top panels) and transgenic strains (bottom panels) were imaged by smFISH using probes hybridizing to the endogenous mRNA of interest (left) and to *mNeonGreen* mRNA (middle) and merged (right). Representative four-cell stage embryos are shown. (B,D) Quantification of images shown in A and C indicating the normalized frequency of *erm-1* (B) or *imb-2* (D) mRNA and *mNeonGreen* mRNA at increasing distances from cell peripheries or nuclear boundaries, respectively, in a single embryo. (F,H) The estimated mRNA content per cluster from a minimum of five embryos at each of five binned stages of development from three biological replicates are reported for endogenous *cpg-2* (F) or *nos-2* (H) (magenta) and *mNeonGreen* reporters (green). *P*-values from multiple test corrected *t*-tests are shown (NS>0.05; 0.05>*>0.005). Scale bars: 10 µm.

3′UTRs of *erm-1* and *imb-2* were not sufficient to drive mRNA subcellular localization. Endogenous *erm-1* and *imb-2* mRNAs localize to the cell or nuclear peripheries, respectively, but *mNeonGreen* mRNA appended with *erm-1* or *imb-2* 3′UTRs failed to recapitulate those patterns (Fig. 4A-D). However, the *imb-2* 3′UTR did show evidence of mRNA destabilization as *Pmex-5::mNeonGreen::imb-2 3*′*UTR* yielded fewer *mNeonGreen* mRNA than endogenous *imb-2* transcripts or *Pmex-5::mNeonGreen::erm-1 3*′*UTR* expressed under the same promoter. This suggests that sequences within the body of the *imb-2* mRNA and/or its successful localization are important for mRNA stability. Ultimately, we did not identify sequences within *erm-1* or *imb-2* mRNAs sufficient to direct transcript localization. Either the 5′ regions of the mRNA, the coding sequence of the mRNA, the full mRNA, a short N-terminal signal peptide or some larger aspect of the translated protein direct mRNA localization.

In contrast, 3′UTRs of *chs-1*, *clu-1*, *cpg-2* and *nos-2* were sufficient to direct *mNeonGreen* mRNA to P granules. Each of the *Pmex-5::mNeonGreen::3*′*UTR-of-interest* strains yielded *mNeonGreen* mRNA localized to P granules coincident with the localization of their endogenous mRNA (Fig. 4E-H, Fig. S7). The *chs-1* 3′UTR did exhibit hallmarks of transcript destabilization given the comparative low abundance of *mNeonGreen::chs-1 3′UTR* transcripts (Fig. S7A).

### RNA localization trends with translational status

NOS-2 protein is translationally repressed in germline and early embryonic stages before becoming translationally active in the P_4_ cell at the 28-cell stage, with both repression and de-repression being mediated by the *nos-2* 3′UTR (D'Agostino et al., 2006). NEONGREEN protein under control of the *nos-2* 3′UTR in our study phenocopied this reported pattern (Fig. S8A). NEONGREEN fused to 3′UTRs of other transcripts (*erm-1*, *imb-2*, *chs-1*, *clu-1* or *cpg-2*) produced low levels of diffuse fluorescence, preventing interpretation of translational status of these reporter transcripts (Fig. S8B).

GFP fusions to full-length ERM-1, CHS-1 and CPG-2 proteins were more informative in illustrating the endogenous expression patterns of the proteins encoded by these localized transcripts. ERM-1::GFP localized to the cell cortex throughout embryogenesis, consistent with the role of the ERM-1 protein in linking the cortical actin cytoskeleton to the plasma membrane (Göbel et al., 2004; Van Fürden et al., 2004) (Fig. S9A). CHS-1 and CPG-2 play a transient role in development, evidenced by GFP fusion reporters showing highest signal in the early cell stages followed by their decline (Fig. S9B,C). CHS-1 and CPG-2 work together to form two different layers of the trilaminar eggshell. CHS-1 encodes a multipass membrane protein that is exocytosed upon fertilization to polymerize chitin (Maruyama et al., 2007; Olson et al., 2012). CHS-1 proteins then internalize, stimulating exocytosis of CPG-1 and CPG-2 proteins that nucleate chondroitin molecules to form the inner eggshell layer – the CPG layer. Indeed, CHS-1::GFP fluoresces at the one-cell stage, but rapidly disappears thereafter (Fig. S9B). CPG-2::GFP appears to be external to the cells and persists within the extracellular space but declines within cells (Fig. S9C). mRNAs encoding both *chs-1* and *cpg-2* cluster in P granules and decline in number as development progresses, as evidenced by our smFISH data. Overall, this shows a trend in which transcripts with repressed, declining or low expression tended to accumulate in P granules.

### Translational repressors of *nos-2* are required for mRNA degradation of multiple transcripts and P granule localization of *nos-2* mRNA

**Fig. 5.**
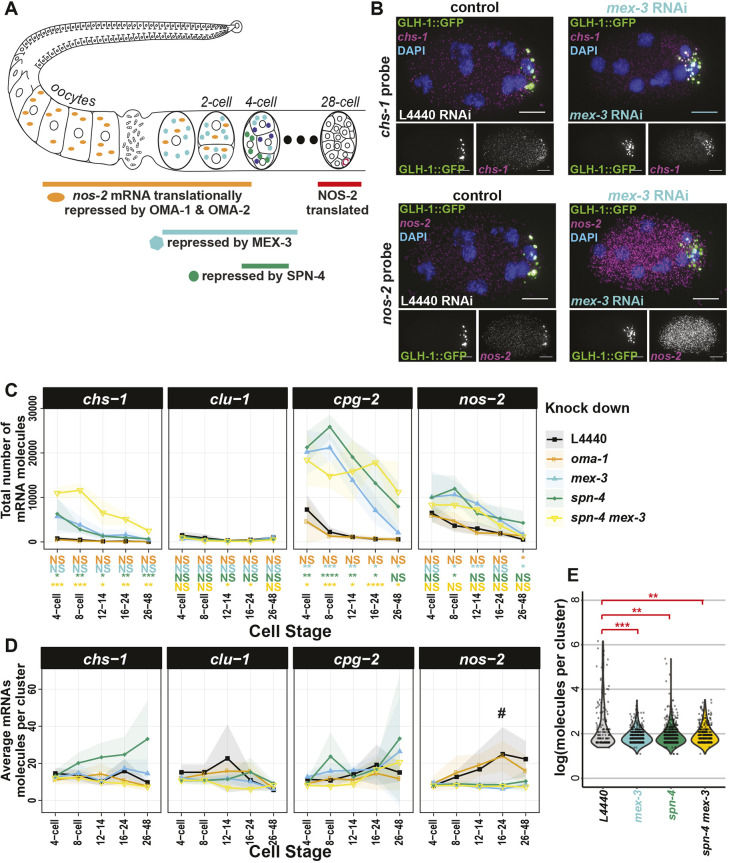
**RBPs that repress translation of *nos-2* mRNA also impact degradation rates and subcellular localization of key mRNAs.** (A) A succession of RBPs cooperatively repress *nos-2* translation from oogenesis through to the 28-cell stage. (B) *chs-1* mRNA (magenta, top) and *nos-2* mRNA (magenta, bottom) were imaged by smFISH in a P granule marker strain (GLH-1::GFP, green) under mock (L4440) and *mex-3* RNAi conditions. (C,D) The total number of mRNA molecules (C) and average number of mRNA molecules per cluster (D) for four different RBP knockdown conditions on five mRNA at five different developmental stages are shown graphically, compared with the L4440 empty vector RNAi control. At least four embryos were assayed for each data point from three biological replicates. Standard deviations are shown as shaded ribbon regions. # indicates data analyzed in E. (E) Distributions of *nos-2* mRNA cluster size under *mex-3*, *spn-4* *(ts)*, and dual *mex-3*/*spn-4* depletion conditions at the 16- to 24-cell stage demonstrate decreased cluster sizes when compared with mock (L4440) depletion. Significance indicates *P*-values derived from multiple test corrected *t*-tests comparing the knockdown condition of interest with vector-only RNAi control (L4440) (0.005>**>0.0005; 0.0005>***>0.00005; 0.00005>****). Scale bars: 10 µm.

*nos-2* is one of three *nanos-*related genes in the *C. elegans* genome and a member of the evolutionarily conserved nanos family. Similar to *Drosophila nanos* mRNA, *C. elegans nos-2* mRNA is contributed maternally, concentrates in the progenitor germ lineage, is translationally repressed in oocytes and during early embryogenesis, is translated with spatial specificity and produces a protein that is expressed only in germ cells (Subramaniam and Seydoux, 1999). *C. elegans nos-2* is required for proper development of the germ cells and is necessary with zygotically-expressed *nos-1* for germ-cell proliferation. Translational repression of *nos-2* is coordinated by four sequential RBPs – OMA-1, OMA-2, MEX-3 and SPN-4 – that directly interact with the *nos-2* 3′UTR (D'Agostino et al., 2006; Jadhav et al., 2008) (Fig. 5A). In oocytes, OMA-1 and OMA-2 are redundantly required to repress translation through direct interactions with the *nos-2* 3′UTR before they are degraded in the zygote. The RBPs MEX-3 and SPN-4 next repress *nos-2* translation throughout the embryo, with SPN-4 being most effective in posterior cells. MEX-3 and SPN-4 both interact with either of two directly repeated RNA sequences in the *nos-2* 3′UTR and function non-redundantly in the early embryo, as RNAi or mutants of either result in premature translation of a *nos-2* reporter. This baton-passing of translational control has been documented for other maternally inherited transcripts including *zif-1* (an E3 ubiquitin ligase specific to somatic cells) (Oldenbroek et al., 2012) and *mom-2* (the Wnt ligand in P_2_) (Oldenbroek et al., 2013).

Though the requirement for OMA-1, OMA-2, MEX-3 and SPN-4 to repress translation of *nos-2* mRNA is clear, owing to a lack of single-molecule resolution FISH data under knockdown conditions it is not known whether they are required to localize *nos-2* mRNA to P granules. To rectify this and to expand the question, we tested how depletion of these RBPs, individually or in combination, impacted the abundance and/or localization of four clustered mRNA transcripts (*chs-1*, *clu-1*, *cpg-2* and *nos-2*) (Fig. 5A). True to published reports, individual knockdowns of OMA-1 and OMA-2 had minimal phenotypes, but in combination yielded too few embryos to credibly test as development arrests during oogenesis (Detwiler et al., 2001; Shimada et al., 2002). Depletion of MEX-3 and/or SPN-4 led to an overabundance of embryo-wide *chs-1*, *cpg-2* and *nos-2* transcripts compared with mock RNAi control, suggesting that MEX-3 and SPN-4 have a direct or indirect role in mRNA degradation (Fig. 5B,C, Fig. S10). MEX-3 and SPN-4 are not required independently to accumulate *chs-1*, *clu-1* or *cpg-2* mRNAs in P granules; however, double knockdown of MEX-3 and SPN-4 resulted in a loss of *chs-1* localization to P granules (Fig. S10)*.* Only the localization of *nos-2* mRNA to P granules was severely disrupted by MEX-3 or SPN-4 loss independently or in combination, as evidenced by the missing *nos-2* clusters in smFISH images (Fig. 5D,E) and corresponding decrease in the average number of mRNA molecules per cluster (Fig. 5C). Together, these findings suggest that MEX-3 and SPN-4 are required for both translational repression and P granule localization of *nos-2* (D'Agostino et al., 2006; Jadhav et al., 2008). Further, the role of MEX-3 and SPN-4 in RNA degradation is separable from their role in mRNA localization to P granules, as *chs-1*, *cpg-2* and *nos-2* require MEX-3 and SPN-4 for RNA clearance, whereas only *nos-2* and *chs-1* rely on them for P granule localization.

### RBPs that relieve NOS-2 translational repression impact *nos-2* localization differently

**Fig. 6.**
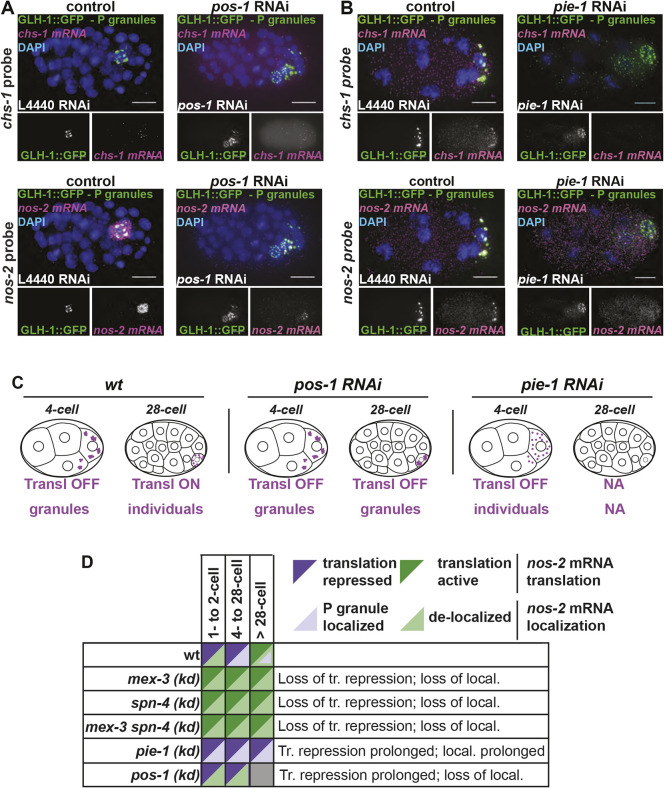
**RBPs that regulate translation of NOS-2 differentially impact *nos-2* mRNA subcellular localization.** (A,B) The impact of depleting POS-1 (A) or PIE-1 (B), two RBPs important for translation activation of *nos-2* mRNA at the 28-cell stage, was assayed. *chs-1* mRNA (magenta, top) and *nos-2* mRNA (magenta, bottom) were imaged in knockdown and control conditions using smFISH in a GLH-1::GFP-expressing strain. DAPI-stained DNA illustrates developmental stage. The 28-cell stage, when *nos-2* normally becomes translationally active, is shown for *pos-1* RNAi conditions. The 8-cell-stage embryo is shown for *pie-1* RNAi conditions to illustrate a stage when *nos-2* is normally repressed. (C) Pictograph demonstrating *nos*-2 behavior under conditions where translation repression is never relieved. (D) Schematic showing a summary of localization and translation phenotypes exhibited in knockdown of *nos*-2 RBPs. Scale bars: 10 μm.

*nos-2* mRNA is translationally repressed in the germline, through fertilization, and is only released from repression at the 28-cell stage of development when NOS-2 protein is exclusively produced in the P_4_ cell (D'Agostino et al., 2006; Subramaniam and Seydoux, 1999; Tenenhaus et al., 2001). *nos-2* mRNA localizes to P granules in the adult germline (Schisa et al., 2001), but appears distinct from P granules at the one- and two-cell stages (this study). Between the four-cell and 28-cell stages, *nos-2* progressively re-accumulates into P granules, reaching a maximum average density of 20-30 mRNA molecules per P granule before the 28-cell stage (Fig. S2, Fig. 2C). At the 28-cell stage of development, when NOS-2 translation begins (Subramaniam and Seydoux, 1999), we observed *nos-2* mRNA becoming dispersed in the cytoplasm external to P granules (Fig. 6A). This could suggest that *nos-2* mRNA emerges from P granules when it becomes actively translated, supported by the fact that P granules are devoid of key ribosomal components required for translation (Schisa et al., 2001).

Because the translational repression of *nos-2* mRNA correlated with its localization to P granules (above, Fig. 5), we sought to determine the effects of prolonged *nos-2* translational repression beyond the 28-cell stage when this repression is typically relieved. We imaged *nos-2* mRNA by smFISH under *pie-1* and *pos-1* RNAi knockdown conditions in which *nos-2* translational repression has been shown to persist (D'Agostino et al., 2006; Tenenhaus et al., 2001). Interestingly, the two knockdown conditions yielded different results. Upon POS-1 depletion, *nos-2* mRNA failed to appear in the cytoplasm after the 28-cell stage and instead remained associated predominantly with P granules (Fig. 6A), as predicted by its translationally inactive status. In contrast, depletion of PIE-1 had the opposite effect. PIE-1 plays a threefold role by contributing to *nos-2* stabilization, NOS-2 translational activation and germline transcriptional repression (D'Agostino et al., 2006; Tenenhaus et al., 2001). Upon disruption of PIE-1, *nos-2* mRNA molecules undergo progressive degradation in the P lineage due to the inappropriate transcription of somatic genes within the P lineage (Seydoux et al., 1996). If this degradation phenotype is abrogated by concurrently blocking somatic gene expression [*pie-1* and *ama-1* (encoding RNA Polymerase II) double knockdown], *nos-2* mRNA molecules survive but fail to produce NOS-2 protein (unlike *ama-1* knockdown alone). The fact that *nos-2* mRNA fails to properly translate after the 28-cell stage under dual *pie-1*/*ama-1* knockdown conditions illustrates that PIE-1 is required to activate the translation of NOS-2 in the P lineage (Tenenhaus et al., 2001). Upon *pie-1* depletion, we confirmed premature *nos-2* mRNA degradation; however, we were surprised to see a complete loss of *nos-2* localization to P granules, despite *nos-2* being translationally inactive at these stages (Tenenhaus et al., 2001) (Fig. 6B). Initially, we suspected that P lineage identity was dysfunctional in these embryos, leading to the loss of wild-type P granule function. However, P granules are clearly present in these embryos (using GLH-1::GFP marker proteins) and they accumulate other mRNAs such as *clu-1* (Fig. 6, Fig. S11). As *nos-2* mRNA is not translated upon *pie-1* disruption (Tenenhaus et al., 2001), this suggests that the translational repression of *nos-2* and its localization to P granules can be uncoupled, perhaps mimicking a somatic-cell-like state in the P lineage.

Taken together, RBP knockdown conditions that disrupt *nos-2* mRNA translational repression also disrupt *nos-2* mRNA P granule association [*mex-3 (RNAi)* and *spn-4 (ts)*] (Fig. 5, Fig. S12). In contrast, an RBP knockdown condition that prolongs *nos-2* translational repression (D'Agostino et al., 2006; Jadhav et al., 2008) fails to release *nos-2* transcripts from P granules [*pos-1 (RNAi)*]. Therefore, the localization of *nos-2* mRNA in P granules is largely coincident with a translationally repressed state (Fig. 6C,D). It is not a perfect association, however. We observed several cases where *nos-2* mRNA remains translationally repressed without localizing to P granules: (1) in one- to two-cell stage embryos; (2) in somatic cells of the early embryo; and (3) in *pie-1* mutants in which *nos-2* fails to localize to P granules (*pie-1* depletion retains *nos-2* repression in Tenenhaus et al., 2001). These findings illustrate that *nos-2* translational repression can occur independently of transcript localization and translational repression is not dependent on P granule residency. Further, it illustrates an order of operations in which translational repression precedes P granule localization during development.

### Disrupting translation promotes P granule localization

**Fig. 7.**
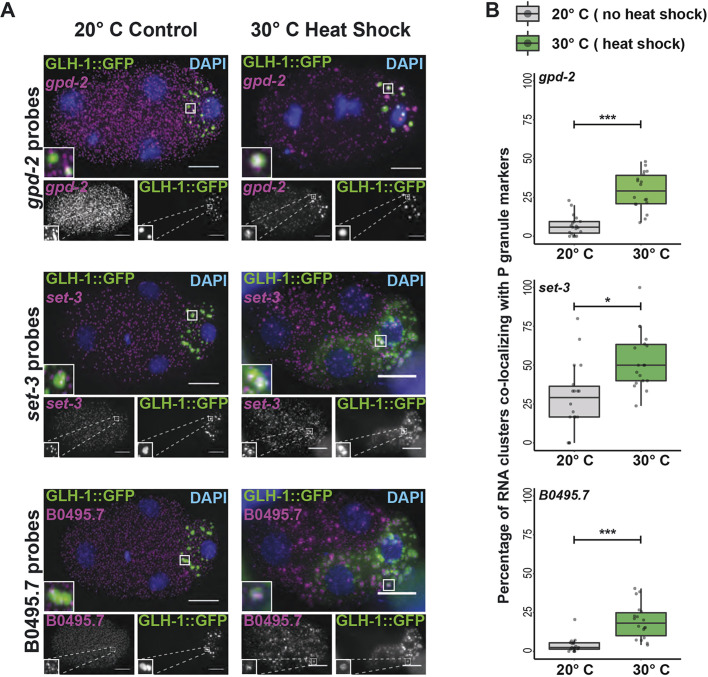
**Homogenously distributed transcripts form clusters when subjected to heat shock stress.** (A) The transcripts *gpd-2*, *set-3* and B0495.7 (magenta) are homogenously distributed in four-cell embryos at 20°C (left). These transcripts become recruited to GLH-1::GFP labeled P granules (green) and other uncharacterized mRNA clusters following a 25 min 30°C heat shock (right). DAPI-stained DNA illustrates developmental stage. Insets show enlarged views of P granules, demonstrating recruitment of RNA to P granules after heat shock. (B) The degree of *gpd-2*, *set-3* and B0495.7 transcript overlap with the P granule marker, GLH-1::GFP, was quantified for embryos cultured at 20°C or heat-shocked at 30°C for 25 min. Box plots show the percentage of RNA clusters overlapping with the P granule marker for each transcript, which was found to significantly increase under heat-shock conditions. Median, and first and third quartile ranges, are indicated by the middle bar and box boundaries, respectively. Whiskers indicate 1.5× the interquartile ranges. All included datapoints are shown as jittered dots. Welch's two sample *t*-test *P*-values are shown: 0.05>*>0.005; 0.005>**>0.0005; ***<0.0005. Scale bars: 10 μm.

We speculated whether P granule localization was a natural consequence that befalls transcripts experiencing low rates of translation or complete repression. To determine whether altering the translational status of mRNAs could change their localization within the cell, we disrupted translational initiation through heat exposure. Embryos exposed to 30°C for 25 min repress protein synthesis at the level of translational initiation (Cuesta et al., 2000; Zevian and Yanowitz, 2014). We observed that three transcripts that are normally homogenously distributed throughout the cytoplasm coalesced into P granules in response to heat stress (Fig. 7, Fig. S13): *set-3*, *gpd-2* (*Glycerol-3-Phosphate Dehydrogenase*) and B0495.7 (predicted metalloprotease). Therefore, loss of protein synthesis was sufficient for otherwise homogenous transcripts to accumulate in P granules.

## DISCUSSION

### Translational repression of mRNA is necessary and sufficient for P granule localization

In this study, we report several maternally inherited mRNAs with subcellular localization in early *C. elegans* embryos. Localization patterns were often associated with translational status. P granule transcripts, for example, had repressed or declining translation. We hypothesized that either mRNAs are actively brought to P granules for the purpose of translational repression, or they are translationally repressed in the cytoplasm leading to their accumulation in P granules. In the case of *nos-2*, three lines of evidence support the second model. First, translational downregulation occurred before P granule localization. Second, in situations where *nos-2* translational repression and P granule localization were uncoupled (one-cell stage, somatic cells and upon *pie-1* depletion), translational repression occurred independently of P granule localization. Finally, heat stress-induced translational repression was sufficient to direct P granule localization. Together, these findings support the model that mRNAs of low translational status accumulate in P granules as a downstream step.

A recent publication by Lee et al. corroborates our findings (Lee et al., 2020). They identified 492 P granule transcripts that precipitate with the intrinsically-disordered P granule factor MEG-3, and they found them to be of low ribosomal occupancy. Indeed, the P granule transcripts they identified depend both on translational repression and on MEG-3 for nucleation into P granules. Loss of P granule association (through *meg-3 meg-4* disruption) did not lead to loss of translational repression. They also illustrated that translational disruption of homogenous transcripts stimulates their ectopic localization into P granules in a MEG-3-dependent manner. Together, our combined works reinforce the interpretation that P granule accumulation occurs as a secondary step preceded and directed by low translational status.

### P granules functionally echo stress granules

mRNAs that localize to P granules could still be observed as individuals within the cytoplasm, as only 7% (*clu-1*, 26- to 48-cell stage) to 53% (*clu-1*, eight-cell stage) of total mRNAs localized to clusters. This echoes stress granules in which 10% of bulk mRNA and up to 95% of specific transcripts move into stress granules only returning to the cytoplasm after the stress has passed (Khong et al., 2017). Though stress granules and germ granules (like P granules) are distinct, they appear to have some functionality in common.

### Different transcripts accumulate in P granules through different mechanisms

We identified six new P granule-enriched transcripts. Of the three (*chs-1*, *clu-1* and *cpg-2*) we selected for further study, all localized to P granules in 3′UTR-dependent manners. However, these transcripts did not rely on the same RBPs for localization into granules as *nos-2* did (MEX-3, SPN-4 and PIE-1). What, then, directs them to P granules? The answer may lie in their biology. CHS-1 and CPG-2 are translationally activated by fertilization but their mRNA and protein levels decline shortly thereafter. Therefore, whether translation is repressed temporarily (*nos-2*) or permanently and followed by degradation (*chs-1* or *cpg-2*), P granule accumulation results*.* Different sets of RBPs likely interpret the 3′UTR sequence information of each transcript to direct regulation.

### mRNA degradation plays a role in shaping transcript localization patterns

Transcripts of *chs-1*, *clu-1*, *cpg-2* and *nos-2* accumulate in the P granules of progenitor germ cells at the same time they disappear from somatic cells. These linked mechanisms concentrate transcripts down the P lineage. All transcripts tested required MEX-3 and SPN-4 for degradation in somatic cells, yet *nos-2* (and to a lesser extent *chs-1* and *cpg-2*) specifically required both RBPs for strong accumulation in P granules. Together, these findings suggest a mechanism in which P granule localization protects mRNAs from MEX-3 and SPN-4-dependent degradation. Local protection coupled to generalized degradation has also been evoked to explain how *Drosophila nanos* concentrates at posterior regions of the embryonic syncytium (Lasko, 2012). Similarly, we found the 3′UTR of *imb-2* fused to *mNeonGreen* elicited *mNeonGreen* mRNA decay, suggesting that *imb-2* localizes to nuclei by a 3′UTR-independent mechanism that protects it from its own 3′UTR-dependent degradation. Together, these findings illustrate how subcellular localization can preserve mRNAs in specific regions of the cell and embryo.

Altogether, translational status directs P granule residency of key transcripts, and P granule residency, in turn, directs enrichment down the P lineage. This explains how mRNAs may be retained and concentrated in specific lineages even in the absence of *de novo* transcription. Indeed, we found that *nos-2* mRNAs within P granules were exceptionally numerous. Whereas other P granule associated transcripts were estimated at 8-12 molecules per granule, *nos-2* mRNAs accumulated to >20 molecules per granule just before the onset of *nos-2* translation. This suggests a possible functional reason why transcripts important for germ cell biology accumulate in P granules – to direct cell-specific protein production even in the absence of *de novo* transcription.

### Peripheral transcripts often encode membrane-associated proteins

Half of the anterior AB-enriched transcripts we surveyed by smFISH accumulated at the cell periphery. Of these, ERM-1 and LEM-3 proteins also localize to apical plasma membranes (Van Fürden et al., 2004; Dittrich et al., 2012). The localizations of APE-1 and TES-1 are currently uncharacterized, but these proteins harbor domains associated with membrane localization (Bennett and Baines, 2001; Sweede et al., 2008). In addition, symmetrically-distributed *imb-2* mRNA localized preferentially at nuclear membranes, the same localization at which the protein it encodes functions (Putker et al., 2013). The concordance between localization of mRNA and the proteins they encode suggest that either the transcripts are directed to membranes for the purpose of local translation or they are passively dragged along behind the growing peptide as it localizes to its final destination. Current genomics assays have illustrated that mRNAs can associate with the endoplasmic reticulum in both translationally-dependent and -independent ways (Chartron et al., 2016), suggesting that both models are possible. Although *erm-1* and *imb-2* lack discernible signal peptides at their N-termini, they both contain membrane-associated domains. Future studies will determine whether these could act to co-translationally direct transcripts to membranes, possibly for the purpose of efficiently generating secondary rounds of translation.

### mRNA localization is a widespread feature of cell biology

Diverse examples of transcript-specific mRNA localization have been described across the tree of life ranging from bacteria (Fei and Sharma, 2018) to humans (Khalil et al., 2018). Although early discoveries of localized mRNAs were thought to represent exceptional cases, recent advances in mRNA proximity labeling suggest that mRNA localization may be more widespread than previously thought (Fazal et al., 2019; Taliaferro, 2019). A new perspective is emerging to encompass mRNA localization control as a general feature of cell biology.

## MATERIALS AND METHODS

### *C. elegans* maintenance

*C. elegans* strains were maintained using standard procedures (Brenner, 1974). Worms were grown at 20°C and reared on nematode growth medium (NGM: 3 g/l NaCl; 17 g/l agar; 2.5 g/l peptone; 5 mg/l cholesterol; 1 mM CaCl_2_; 1 mM MgSO_4_; 2.7 g/l KH_2_PO_4_; 0.89 g/l K_2_HPO_4_). *C. elegans* strains generated in this study were derived from the standard laboratory strain, Bristol N2. Strains used in this study are listed in Table S1.

### Ethics and oversight

All experiments were subject to oversight by the Colorado State University Institutional Biosafety Committee and were conducted in accordance with National Institutes of Health guidelines.

### 3′UTR reporter constructs

The plasmid pMTNCSU7 was generated to express mNeonGreen as an N-terminal fluorescent reporter. Starting with a *Pmex-5::neongreen::neg-1*::*neg-1*-3′UTR plasmid derived from the MosSCI-based plasmid pCFJ150, we replaced the *neg-1* sequences with an NheI/BglII/EcoRV multiple cloning site using inverse PCR. 3′UTRs were PCR amplified and cloned into the NheI site of pMTNCSU7 using Gibson cloning (New England Biolabs) to create pDMP45 (*Pmex-5::mNeonGreen::nos-2 3′UTR*), pDMP47 (*Pmex-5::mNeonGreen::cpg-2 3′UTR*), pDMP48 (*Pmex-5::mNeonGreen::chs-1 3′UTR*), pDMP91 (*Pmex-5::mNeonGreen::clu-1 3′UTR*), pDMP111 (*Pmex-5::mNeonGreen::imb-2 3′UTR*) and pDMP112 (*Pmex-5::mNeonGreen::erm-1 3′UTR*). Plasmids used in this study are listed in Table S2. Primers used for 3′UTR amplification can be found in Table S3.

### *C. elegans* single-copy transgenesis by CRISPR

*Pmex-5::mNeonGreen::3′UTR* strains were generated from N2 worms by CRISPR targeting to the ttTi5605 MosSCI site (Dickinson et al., 2013). Guide RNA targeting the ttTi5605 MosSCI site and Cas9 protein were co-expressed from the plasmid pDD122, whereas plasmids pDMP45, pDMP47, pDMP48, pDMP91, pDMP111 and pDMP112 were used as repair templates. Three vectors containing mCherry-tagged pGH8 (*Prab-8::mCherry* neuronal co-injection marker), pCFJ104 (*Pmyo-3::mCherry* body wall muscle co-injection marker) and pCFJ90 (*Pmyo-2::mCherry* pharyngeal co-injection marker) as well as one containing the heat-shock activated PEEL-1 counter-selectable marker (pMA122) were co-injected. mNeonGreen- and mCherry-positive animals were identified as F1 progeny and singled to new plates until starvation. Starved plates were then subjected to a 4 h incubation at 34°C to counterselect, followed by an overnight recovery at 25°C. Plates were then screened for living worms that did not express the mCherry co-injection markers. Worms that showed no fluorescence from the presence of extrachromosomal arrays were singled to establish lines, which were confirmed for single-copy insertion by PCR using the primers in Table S3.

### smFISH

smFISH was performed based on the TurboFish protocol, with updates specific to *C. elegans* and using new Biosearch reagents (Femino et al., 1998; Osborne Nishimura et al., 2015; Raj and Tyagi, 2010; Raj et al., 2008; Shaffer et al., 2013). Custom Stellaris FISH Probes were designed against target transcripts (Table S4) using the Stellaris RNA FISH Probe Designer (Biosearch Technologies; www.biosearchtech.com/stellarisdesigner; version 4.2). The embryos were hybridized with Stellaris RNA FISH Probe sets labeled with CalFluor 610 or Quasar 670 (Biosearch Technologies) following the manufacturer's instructions (www.biosearchtech.com/stellarisprotocols). Briefly, young adult worms were bleached for embryos, suspended in 1 ml −20°C methanol, quickly vortexed and freeze cracked in liquid nitrogen. Embryos were stored in methanol at −20°C for 1-24 h. After fixation, embryos were equilibrated briefly in Stellaris Wash Buffer A (Biosearch Technologies, SMF-WA1-60) before hybridization in 100 µl Stellaris Hybridization buffer (Biosearch Technologies, SMF-HB1-10) containing 10% formamide and 50 pmol of each primer set. The hybridization reaction was incubated at 37°C overnight. Hybridized embryos were then washed twice for 30 min in Stellaris Wash Buffer A, with the second wash containing 1 µg/ml of DAPI. Following counterstaining, a final wash in Stellaris Wash Buffer B (Biosearch Technologies, SMF-WB1-20) was carried out before storage with N-propyl gallate antifade [10 ml 100% glycerol, 100 mg N-propyl gallate, 400 µl 1 M Tris (pH 8.0), 9.6 ml DEPC-treated H_2_O] before slide preparation. Embryos were mounted based on original descriptions in Ji and van Oudenaarden (2012), using equal volumes of hybridized embryos resuspended in N-propyl gallate antifade and Vectashield antifade (Vector Laboratories, H-1000). smFISH image stacks were acquired on a Photometrics Cool Snap HQ2 camera using a DeltaVision Elite inverted microscope (GE Healthcare), with an Olympus PLAN APO 60× (1.42 NA, PLAPON60XOSC2) objective, an Insight SSI 7-Color Solid State Light Engine and SoftWorx software (Applied Precision) using 0.2 µm *z*-stacks. Representative images were deconvolved using Deltavision (SoftWorx) deconvolution software. Images were further processed using FIJI (Schindelin et al., 2012). Initial characterization of subcellular localization for the transcripts *erm-1*, *imb-2*, *chs-1*, *clu-1*, *cpg-2* and *nos-2* was performed in conjunction with the homogenous transcript *set-3* as a negative control for subcellular localization (data not shown; see http://dx.doi.org/10.25675/10217/201623 for raw microscopy images). In all instances, a minimum of five embryos, but often many more, were imaged for each genetic condition and time point. All raw microscopy images are deposited on Mountain Scholar, a digital, open access data repository associated with Colorado State University Libraries (http://dx.doi.org/10.25675/10217/201623).

### smiFISH

smiFISH was performed as in Tsanov et al. (2016) using FLAPY primary probe extensions and secondary probes. Briefly, between 12 and 24 primary probes were designed using Oligostan (Tsanov et al., 2016) and ordered in 25 nmol 96-well format from Integrated DNA Technologies diluted to 100 µM in IDTE buffer (pH 8.0). Secondary FLAPY probes were ordered from Stellaris LGC with dual 5′ and 3′ fluorophore labeling using either Cal Fluor 610 or Quasar 670 (Biosearch Technologies, BNS-5082 and FC-1065, respectively). Individual probes were combined to a final concentration of 0.833 µM, and 2 µl of primary probe mixture were mixed with 1 µl 50 µM FLAPY secondary probe, 1 µl NEB buffer 3 and 6 µl DEPC-treated H_2_O. The primary and secondary probe mixtures were then incubated in a thermocycler at 85°C for 3 min, 65°C for 3 min and 25°C for 5 min to anneal. Then 2 µl of annealed probe mixtures were used as normal smFISH probe sets as above. smiFISH probe sequences are listed in Table S4.

### smFISH plus immunofluorescence

smFISH combined with immunofluorescence was performed similarly to smFISH with slight modifications. N2 and DUP98 *patr-1(sam50[patr-1::GFP::3xFLAG])II* (Andralojc et al., 2017) embryos were harvested as above with the exception that they were resuspended in methanol, freeze cracked in liquid nitrogen for 1 min, and transferred to acetone after ∼5 min total in methanol. Embryos were then incubated in acetone for 25 min before proceeding to hybridization/immunofluorescence. smFISH was then performed as above with the exception that a final concentration of 2.37 µg/ml Janelia Fluor 549 (Tocris, 6147) conjugated anti-GFP nanobody (Chromotek, gt-250) was incubated with the embryos overnight in hybridization buffer.

### Initial quantification of smFISH micrographs

Initial characterization of mRNA counts from smFISH micrographs was performed using a standard FISH-quant (Mueller et al., 2013) analysis. Briefly, embryos were manually outlined, 3D LoG filtered using default FISH-quant parameters (size=5, s.d.=1), spots were pre-detected using a local maximum fitting and RNAs were detected using a manually determined image-dependent intensity and quality threshold, with sub-region fitting of 2 pixels in the *x*- and *y*-axes and 3 pixels in the *z*-axis.

Post-processing to calculate the different location metrics was performed as described below with custom-written Matlab and Python code. The Python code is implemented as plugins for the image processing platform ImJoy (Ouyang et al., 2019 preprint). Source code and detailed description are provided at https://github.com/muellerflorian/parker-rna-loc-elegans.

### Quantification of cortical RNA localization

Quantification of transcript localization to the cell cortex was performed using the web application ImJoy (Ouyang et al., 2019 preprint). RNAs were first detected as above using FISH-quant. Individual cell outlines were then manually annotated in FIJI for each *z*-stack in the micrograph, excluding the uppermost and lowermost stacks where cells are flattened against the slide or coverslip. The distance of each RNA was then measured from the nearest annotated membrane and binned in 10 µm increments. The total number of RNAs per bin was then normalized by the volume of the concentric spheres they occupied. After this normalization, values larger than 1 indicate that for this distance more RNAs are found compared with a randomly distributed sample.

### Quantification of nuclear peripheral RNA localization

Quantification of transcript localization to the nuclear periphery was also performed using ImJoy. RNAs were first detected as above using FISH-quant. Embryos were then manually outlined to create an upper limit for RNA distance from the nucleus. Individual nuclei were then annotated by binarizing DAPI micrographs to create a nuclear mask. The distance of each RNA was then measured from the nearest annotated nuclear membrane and binned in 10 µm increments. Negative distance indicates positioning within the nuclear mask. The total number of RNAs per bin was then normalized for volume as described above for cell membrane localization.

### Quantification of RNA clustering

Detection of RNA molecules was performed in the 3D image stacks using FISH-quant (Mueller et al., 2013). Positions of individual RNA molecules within dense clusters were determined with a recently developed approach using the signal of isolated RNAs to decompose these clusters (Samacoits et al., 2018). Post-processing to calculate the different location metrics was performed as described below with custom-written Matlab and Python code. The Python code is implemented in user-friendly plugins for the image processing platform ImJoy (Ouyang et al., 2019 preprint). Source code and all scripts used for analysis and figure generation are available at https://github.com/muellerflorian/parker-rna-loc-elegans.

To quantify the number of individual mRNAs in mRNA clusters, the total number of clusters per embryo and the fraction of mRNAs in clusters, a custom MATLAB script was implemented. FISH-quant detection settings were used to identify candidate mRNA clusters from smFISH micrographs using GMM. The GMM differentiates independent, single mRNAs from groups of clustered mRNAs by probabilistically fitting a predicted RNA of average intensity and size over each FISH-quant detected RNA. GMM fitting then provided coordinates of both independent RNAs and the modeled coordinates of each RNA that composes a cluster. The decomposed coordinates of each RNA in the embryo were then used by a density-based spatial clustering of applications with noise (DBSCAN) algorithm to quantitatively analyze cluster size and number.

### Quantifying RNA cluster overlap with GLH-1::GFP

To determine the degree of overlap between RNA clusters and P granules labeled with GLH-1::GFP a hybrid Matlab-ImJoy pipeline was implemented. RNA clusters were identified as described above. The occupied volume of these clusters in the image was calculated as the convex hull around all RNA positions within a cluster with the SciPy function ConvexHull. The location of P granules was determined in 3D with a Laplacian of Gaussian (LoG) blob detection method (with the scikit-image function blog_log). RNA clusters and P granules were considered to co-localize when their 3D volumes at least partly overlap. This allowed quantification of the number of independent P granules, RNA clusters, and RNA clusters that overlap with P granules.

### RNAi feeding for smFISH microscopy

dsRNA feeding was executed as previously described (Sawyer et al., 2011). Mixed-stage worms were bleached to harvest and synchronize embryos. Harvested embryos were deposited on RNAi feeding plates and grown at 25°C until gravid. Embryos were harvested and smFISH was conducted. For each gene targeted by RNAi, we performed at least three independent replicates of feeding and smFISH using L4440 empty vector as a negative control and *pop-1* RNAi as a 100% embryonic lethal positive control. For experiments using the *spn-4* temperature sensitive allele, *spn-4(or191) V*, worms were grown at 15°C until gravid, bleached for embryos, and split into 15°C negative control and 25°C query conditions while plating on L4440, *mex-3* or *pop-1* RNAi conditions.

## Supplementary Material

Supplementary information

Reviewer comments

## References

[DEV186817C1] Andralojc K. M., Campbell A. C., Kelly A. L., Terrey M., Tanner P. C., Gans I. M., Senter-Zapata M. J., Khokhar E. S. and Updike D. L. (2017). ELLI-1, a novel germline protein, modulates RNAi activity and P-granule accumulation in Caenorhabditis elegans. *PLoS Genet.* 13, 1-20. 10.1371/journal.pgen.1006611 PMC5325599 28182654

[DEV186817C2] Bennett V. and Baines A. J. (2001). Spectrin and ankyrin-based pathways: metazoan inventions for integrating cells into tissues. *Physiol. Rev.* 81, 1353-1392. 10.1152/physrev.2001.81.3.1353 11427698

[DEV186817C3] Brangwynne C. P., Eckmann C. R., Courson D. S., Rybarska A., Hoege C., Gharakhani J., Jülicher F. and Hyman A. A. (2009). Germline P granules are liquid droplets that localize by controlled dissolution/condensation. *Science (80-. )* 5, 1729-1732. 10.1126/science.1172046 19460965

[DEV186817C4] Brenner S. (1974). The genetics of Caenorhabditis elegans. *Genetics* 77, 71-94. 4366476 10.1093/genetics/77.1.71 PMC1213120

[DEV186817C5] Campbell A. C. and Updike D. L. (2015). CSR-1 and P granules suppress sperm-specific transcription in the C. elegans germline. *Development* 142, 1745-1755. 10.1242/dev.121434 25968310 PMC4440928

[DEV186817C6] Chartron J. W., Hunt K. C. L. and Frydman J. (2016). Cotranslational signal-independent SRP preloading during membrane targeting. *Nature* 536, 224-228. 10.1038/nature19309 27487213 PMC5120976

[DEV186817C7] Cuesta R., Laroia G. and Schneider R. J. (2000). Chaperone Hsp27 inhibits translation during heat shock by binding eIF4G and facilitating dissociation of cap-initiation complexes. *Genes Dev.* 14, 1460-1470. 10859165 PMC316692

[DEV186817C8] D'Agostino I., Merritt C., Chen P., Seydoux G. and Subramaniam K. (2006). Translational repression restricts expression of the C. elegans Nanos homolog NOS-2 to the embryonic germline*. Dev. Biol.* 292, 244-252. 10.1016/j.ydbio.2005.11.046 16499902

[DEV186817C9] DeRenzo C., Reese K. J. and Seydoux G. (2003). Exclusion of germ plasm proteins from somatic lineages by cullin-dependent degradation. *Nature* 424, 685-689. 10.1038/nature01887 12894212 PMC1892537

[DEV186817C10] Detwiler M. R., Reuben M., Li X., Rogers E. and Lin R. (2001). Two zinc finger proteins, OMA-1 and OMA-2, are redundantly required for oocyte maturation in C. elegans. *Dev. Cell* 1, 187-199. 10.1016/S1534-5807(01)00026-0 11702779

[DEV186817C11] Dickinson D. J., Ward J. D., Reiner D. J. and Goldstein B. (2013). Engineering the Caenorhabditis elegans genome using Cas9-triggered homologous recombination. *Nat. Methods* 10, 1028-1034. 10.1038/nmeth.2641 23995389 PMC3905680

[DEV186817C12] Dittrich C. M., Kratz K., Sendoel A., Gruenbaum Y., Jiricny J. and Hengartner M. O. (2012). Lem-3 - a lem domain containing nuclease involved in the dna damage response in c. elegans. *PLoS ONE* 7, e24555 10.1371/journal.pone.0024555 22383942 PMC3285610

[DEV186817C13] Eagle W. V. I., Yeboah-Kordieh D. K., Niepielko M. G. and Gavis E. R. (2018). Distinct cis-acting elements mediate targeting and clustering of drosophila polar granule mRNAs. *Development* 145, dev164657 10.1242/dev.164657 30333216 PMC6262787

[DEV186817C14] Elewa A., Shirayama M., Kaymak E., Harrison P. F., Powell D. R., Du Z., Chute C. D., Woolf H., Yi D., Ishidate T. et al. (2015). POS-1 promotes endo-mesoderm development by inhibiting the Cytoplasmic polyadenylation of neg-1 mRNA. *Dev. Cell* 34, 108-118. 10.1016/j.devcel.2015.05.024 26096734 PMC4507413

[DEV186817C15] Ester M., Kriegel H.-P., Sander J. and Xu X. (1996). A density-based algorithm for discovering clusters in large spatial databases with noise. In Proceedings of the 2nd International Conference on Knowledge Discovery and Data Mining, pp. 226-231.

[DEV186817C16] Fazal F. M., Han S., Parker K. R., Kaewsapsak P., Xu J., Boettiger A. N., Chang H. Y. and Ting A. Y. (2019). Atlas of subcellular RNA localization revealed by APEX-Seq. *Cell* 178, 473-490.e26. 10.1016/j.cell.2019.05.027 31230715 PMC6786773

[DEV186817C81] Fei J. and Sharma C. M. (2018). RNA Localization in Bacteria. *Microbiol. Spectr*. 6, 10.1128/microbiolspec.RWR-0024-2018 10.1128/microbiolspec.RWR-0024-2018 PMC6264921 30191804

[DEV186817C17] Femino A. M., Fay F. S., Fogarty K. and Singer R. H. (1998). Visualization of single RNA transcripts in situ. *Science (80-. )* 280, 585-590. 10.1126/science.280.5363.585 9554849

[DEV186817C18] Fields S. D., Conrad M. N. and Clarke M. (1998). The S. cerevisiae CLU1 and D. discoideum cluA genes are functional homologues that influence mitochondrial morphology and distribution. *J. Cell Sci.* 111, 1717-1727. 9601101 10.1242/jcs.111.12.1717

[DEV186817C19] Gallo C. M., Munro E., Rasoloson D., Merritt C. and Seydoux G. (2008). Processing bodies and germ granules are distinct RNA granules that interact in C. elegans embryos. *Dev. Biol.* 323, 76-87. 10.1016/j.ydbio.2008.07.008 18692039

[DEV186817C20] Gallo C. M., Wang J. T., Motegi F. and Seydoux G. (2010). Cytoplasmic partitioning of P granule components is not required to specify the germline in C. elegans. *Science* 330, 1685-1689. 10.1126/science.1193697 21127218 PMC3072820

[DEV186817C21] Gibert M. A., Starck J. and Beguet B. (1984). Role of the gonad cytoplasmic core during oogenesis of the nematode Caenorhabditis elegans. *Biol. Cell* 50, 77-85. 10.1111/j.1768-322X.1984.tb00254.x 6234041

[DEV186817C22] Göbel V., Barrett P. L., Hall D. H. and Fleming J. T. (2004). Lumen morphogenesis in C. elegans requires the membrane-cytoskeleton linker erm-1. *Dev. Cell* 6, 865-873. 10.1016/j.devcel.2004.05.018 15177034

[DEV186817C23] Guven-Ozkan T., Robertson S. M., Nishi Y. and Lin R. (2010). zif-1 translational repression defines a second, mutually exclusive OMA function in germline transcriptional repression. *Development* 137, 3373-3382. 10.1242/dev.055327 20826530 PMC2947753

[DEV186817C24] Hamm D. C. and Harrison M. M. (2018). Regulatory principles governing the maternal-to-zygotic transition: insights from Drosophila melanogaster. *Open Biol.* 8, 180183 10.1098/rsob.180183 30977698 PMC6303782

[DEV186817C25] Hashimshony T., Wagner F., Sher N. and Yanai I. (2012). CEL-Seq: single-cell RNA-Seq by multiplexed linear amplification. *Cell Rep.* 2, 666-673. 10.1016/j.celrep.2012.08.003 22939981

[DEV186817C26] Hashimshony T., Feder M., Levin M., Hall B. K. and Yanai I. (2015). Spatiotemporal transcriptomics reveals the evolutionary history of the endoderm germ layer. *Nature* 519, 219-222. 10.1038/nature13996 25487147 PMC4359913

[DEV186817C27] Hird S. N., Paulsen J. E. and Strome S. (1996). Segregation of germ granules in living Caenorhabditis elegans embryos: Cell-type-specific mechanisms for cytoplasmic localisation. *Development* 122, 1303-1312. 8620857 10.1242/dev.122.4.1303

[DEV186817C28] Jadhav S., Rana M. and Subramaniam K. (2008). Multiple maternal proteins coordinate to restrict the translation of C. elegans nanos-2 to primordial germ cells. *Development* 135, 1803-1812. 10.1242/dev.013656 18417623 PMC2573031

[DEV186817C29] Ji N. and van Oudenaarden A. (2012). Single molecule fluorescent in situ hybridization (smFISH) of C. elegans worms and embryos. *WormBook* 1-16. 10.1895/wormbook.1.153.1 PMC4781154 23242966

[DEV186817C30] Kawasaki I., Amiri A., Fan Y., Meyer N., Dunkelbarger S., Motohashi T., Karashima T., Bossinger O. and Strome S. (2004). The PGL family proteins associate with germ granules and function redundantly in Caenorhabditis elegans germline development. *Genetics* 167, 645-661. 10.1534/genetics.103.023093 15238518 PMC1470885

[DEV186817C82] Khalil B., Morderer D., Price P. L., Liu F. and Rossoll W (2018). mRNP assembly, axonal transport, and local translation in neurodegenerative diseases. *Brain Res*. 1693, 75-91. 10.1016/j.brainres.2018.02.018 29462608 PMC5997521

[DEV186817C31] Khong A., Matheny T., Jain S., Mitchell S. F., Wheeler J. R. and Parker R. (2017). The stress granule transcriptome reveals principles of mRNA accumulation in stress granules. *Mol. Cell* 68, 808-820.e5. 10.1016/j.molcel.2017.10.015 29129640 PMC5728175

[DEV186817C32] Lasko P. (2012). mRNA localization and translational control in Drosophila oogenesis. *Cold Spring Harb. Perspect. Biol.* 4, a012294 10.1101/cshperspect.a012294 22865893 PMC3475173

[DEV186817C33] Lee C. Y. S., Putnam A., Lu T., He S., Ouyang J. P. T. and Seydoux G. (2020). Recruitment of mRNAs to P granules by condensation with intrinsically-disordered proteins. *Elife* 9, e52896 10.7554/eLife.52896 31975687 PMC7007223

[DEV186817C34] Marnik E. A. and Updike D. L. (2019). Membraneless organelles: P granules in Caenorhabditis elegans. *Traffic* 20, 373-379. 10.1111/tra.12644 30924287 PMC6571499

[DEV186817C35] Martin K. C. and Ephrussi A. (2009). mRNA Localization: gene expression in the spatial dimension. *Cell* 136, 719-730. 10.1016/j.cell.2009.01.044 19239891 PMC2819924

[DEV186817C36] Maruyama R., Velarde N. V., Klancer R., Gordon S., Kadandale P., Parry J. M., Hang J. S., Rubin J., Stewart-Michaelis A., Schweinsberg P. et al. (2007). EGG-3 regulates cell-surface and cortex rearrangements during egg activation in caenorhabditis elegans. *Curr. Biol.* 17, 1555-1560. 10.1016/j.cub.2007.08.011 17869112

[DEV186817C37] Merritt C., Rasoloson D., Ko D. and Seydoux G. (2008). 3′ UTRs are the primary regulators of gene expression in the C. elegans germline. *Curr. Biol.* 18, 1476-1482. 10.1016/j.cub.2008.08.013 18818082 PMC2585380

[DEV186817C38] Mueller F., Senecal A., Tantale K., Marie-Nelly H., Ly N., Collin O., Basyuk E., Bertrand E., Darzacq X. and Zimmer C. (2013). FISH-quant: automatic counting of transcripts in 3D FISH images. *Nat. Methods* 10, 277-278. 10.1038/nmeth.2406 23538861

[DEV186817C39] Oldenbroek M., Robertson S. M., Guven-Ozkan T., Gore S., Nishi Y. and Lin R. (2012). Multiple RNA-binding proteins function combinatorially to control the soma-restricted expression pattern of the E3 ligase subunit ZIF-1. *Dev. Biol.* 363, 388-398. 10.1016/j.ydbio.2012.01.002 22265679 PMC5873315

[DEV186817C40] Oldenbroek M., Robertson S. M., Guven-Ozkan T., Spike C., Greenstein D. and Lin R. (2013). Regulation of maternal Wnt mRNA translation in C. elegans embryos. *Development* 140, 4614-4623. 10.1242/dev.096313 24131629 PMC3817945

[DEV186817C41] Olson S. K., Greenan G., Desai A., Müller-Reichert T. and Oegema K. (2012). Hierarchical assembly of the eggshell and permeability barrier in C. Elegans. *J. Cell Biol.* 198, 731-748. 10.1083/jcb.201206008 22908315 PMC3514041

[DEV186817C42] Osborne Nishimura E., Zhang J. C., Werts A. D., Goldstein B. and Lieb J. D. (2015). Asymmetric transcript discovery by RNA-seq in C. elegans blastomeres identifies neg-1, a gene important for anterior morphogenesis*. PLoS Genet.* 11, 1-29. 10.1371/journal.pgen.1005117 PMC4395330 25875092

[DEV186817C43] Ouyang W., Mueller F., Hjelmare M., Lundberg E. and Zimmer C. (2019). ImJoy: an open-source computational platform for the deep learning era. *Nat. Methods* **16**, 1199-1200 10.1038/s41592-019-0627-0 31780825

[DEV186817C44] Parker R. and Sheth U. (2007). P Bodies and the control of mRNA translation and degradation. *Mol. Cell* 25, 635-646. 10.1016/j.molcel.2007.02.011 17349952

[DEV186817C45] Phillips C. M., Montgomery T. A., Breen P. C. and Ruvkun G. (2012). MUT-16 promotes formation of perinuclear Mutator foci required for RNA silencing in the C. elegans germline. *Genes Dev.* 26, 1433-1444. 10.1101/gad.193904.112 22713602 PMC3403012

[DEV186817C46] Putker M., Madl T., Vos H. R., de Ruiter H., Visscher M., van den Berg M. C. W., Kaplan M., Korswagen H. C., Boelens R., Vermeulen M. et al. (2013). Redox-dependent control of FOXO/DAF-16 by transportin-1. *Mol. Cell* 49, 730-742. 10.1016/j.molcel.2012.12.014 23333309

[DEV186817C47] Raj A. and Tyagi S. (2010). *Detection of Individual Endogenous RNA Transcripts in Situ Using Multiple Singly Labeled Probes*, 1st edn Elsevier Inc. 10.1016/S0076-6879(10)72004-8 20580972

[DEV186817C48] Raj A., van den Bogaard P., Rifkin S. A., van Oudenaarden A. and Tyagi S. (2008). Imaging individual mRNA molecules using multiple singly labeled probes. *Nat. Methods* 5, 877-879. 10.1038/nmeth.1253 18806792 PMC3126653

[DEV186817C49] Robertson S. and Lin R. (2015). *The Maternal-to-Zygotic Transition in C. elegans*, 1st edn Elsevier Inc. 10.1016/bs.ctdb.2015.06.001 26358869

[DEV186817C50] Samacoits A., Chouaib R., Safieddine A., Traboulsi A. M., Ouyang W., Zimmer C., Peter M., Bertrand E., Walter T. and Mueller F. (2018). A computational framework to study sub-cellular RNA localization. *Nat. Commun.* 9, 4584 10.1038/s41467-018-06868-w 30389932 PMC6214940

[DEV186817C51] Sawyer J. M., Glass S., Li T., Shemer G., White N. D., Starostina N. G., Kipreos E. T., Jones C. D. and Goldstein B. (2011). Overcoming redundancy: an RNAi enhancer screen for morphogenesis genes in caenorhabditis elegans. *Genetics* 188, 549-564. 10.1534/genetics.111.129486 21527776 PMC3176534

[DEV186817C52] Schindelin J., Arganda-Carreras I., Frise E., Kaynig V., Longair M., Pietzsch T., Preibisch S., Rueden C., Saalfeld S., Schmid B. et al. (2012). Fiji: an open-source platform for biological-image analysis. *Nat. Methods* 9, 676-682. 10.1038/nmeth.2019 22743772 PMC3855844

[DEV186817C53] Schisa J. A., Pitt J. N. and Priess J. R. (2001). Analysis of RNA associated with P granules in germ cells of C. elegans adults. *Development* 128, 1287-1298. 11262230 10.1242/dev.128.8.1287

[DEV186817C54] Schulz K. N. and Harrison M. M. (2019). Mechanisms regulating zygotic genome activation. *Nat. Rev. Genet.* 20, 221-234. 10.1038/s41576-018-0087-x 30573849 PMC6558659

[DEV186817C55] Seydoux G. (2018). The P Granules of C. elegans: a genetic model for the study of RNA–protein condensates. *J. Mol. Biol.* 430, 4702-4710. 10.1016/j.jmb.2018.08.007 30096346 PMC6779309

[DEV186817C56] Seydoux G. and Fire A. (1994). Soma-germline asymmetry in the distributions of embryonic RNAs in Caenorhabditis elegans. *Development* 120, 2823-2834. 7607073 10.1242/dev.120.10.2823

[DEV186817C57] Seydoux G., Mello C. C., Pettitt J., Wood W. B., Priess J. R. and Fire A. (1996). Repression of gene expression in the embryonic germ lineage of C. elegans. *Nature* 382, 713-716. 10.1038/382713a0 8751441

[DEV186817C58] Shaffer S. M., Wu M. T., Levesque M. J. and Raj A. (2013). Turbo FISH: a method for rapid single molecule RNA fish. *PLoS ONE* 8, e75120 10.1371/journal.pone.0075120 24066168 PMC3774626

[DEV186817C59] Sheth U., Pitt J., Dennis S. and Priess J. R. (2010). Perinuclear P granules are the principal sites of mRNA export in adult C. elegans germ cells. *Development* 137, 1305-1314. 10.1242/dev.044255 20223759 PMC2847466

[DEV186817C60] Shimada M., Kawahara H. and Doi H. (2002). Novel family of CCCH-type zinc-finger proteins, MOE-1, −2 and −3, participates in C. elegans oocyte maturation. *Genes Cells* 7, 933-947. 10.1046/j.1365-2443.2002.00570.x 12296824

[DEV186817C61] Spike C. A., Coetzee D., Nishi Y., Guven-Ozkan T., Oldenbroek M., Yamamoto I., Lin R. and Greenstein D. (2014). Translational control of the oogenic program by components of OMA ribonucleoprotein particles in caenorhabditis elegans. *Genetics* 198, 1513-1533. 10.1534/genetics.114.168823 25261697 PMC4256769

[DEV186817C62] Strome S. and Wood W. B. (1982). Immunofluorescence visualization of germ-line-specific cytoplasmic granules in embryos, larvae, and adults of Caenorhabditis elegans. *Proc. Natl. Acad. Sci. USA* 79, 1558-1562. 10.1073/pnas.79.5.1558 7041123 PMC346014

[DEV186817C63] Subramaniam K. and Seydoux G. (1999). nos-1 and nos-2, two genes related to Drosophila nanos, regulate primordial germ cell development and survival in Caenorhabditis elegans. *Development* 4871, 4861-4871. 10.1242/dev.126.21.4861 10518502

[DEV186817C64] Sweede M., Ankem G., Chutvirasakul B., Azurmendi H. F., Chbeir S., Watkins J., Helm R. F., Finkielstein C. V. and Capelluto D. G. S. (2008). Structural and membrane binding properties of the prickle PET domain. *Biochemistry* 47, 13524-13536. 10.1021/bi801037h 19053268

[DEV186817C65] Taliaferro J. M. (2019). Classical and emerging techniques to identify and quantify localized RNAs. *Wiley Interdiscip. Rev. RNA* 10, e1542 10.1002/wrna.1542 31044542

[DEV186817C66] Tenenhaus C., Subramaniam K., Dunn M. A. and Seydoux G. (2001). PIE-1 is a bifunctional protein that regulates maternal and zygotic gene expression in the embryonic germ line of Caenorhabditis elegans. *Genes Dev.* 15, 1031-1040. 10.1101/gad.876201 11316796 PMC312670

[DEV186817C67] Tintori S. C., Osborne Nishimura E., Golden P., Lieb J. D. and Goldstein B. (2016). A transcriptional lineage of the early C. elegans embryo. *Dev. Cell* 38, 430-444. 10.1016/j.devcel.2016.07.025 27554860 PMC4999266

[DEV186817C68] Trcek T., Grosch M., York A., Shroff H., Lionnet T. and Lehmann R. (2015). Drosophila germ granules are structured and contain homotypic mRNA clusters. *Nat. Commun.* 6, 7962 10.1038/ncomms8962 26242323 PMC4918342

[DEV186817C69] Tsanov N., Samacoits A., Chouaib R., Traboulsi A. M., Gostan T., Weber C., Zimmer C., Zibara K., Walter T., Peter M. et al. (2016). SmiFISH and FISH-quant - A flexible single RNA detection approach with super-resolution capability. *Nucleic Acids Res.* 44, e165 10.1093/nar/gkw784 27599845 PMC5159540

[DEV186817C70] Updike D. L., Knutson A. K. A., Egelhofer T. A., Campbell A. C. and Strome S. (2014). Germ-granule components prevent somatic development in the C. Elegans germline. *Curr. Biol.* 24, 970-975. 10.1016/j.cub.2014.03.015 24746798 PMC4036631

[DEV186817C71] Van Fürden D., Johnson K., Segbert C. and Bossinger O. (2004). The C. elegans ezrin-radixin-moesin protein ERM-1 is necessary for apical junction remodelling and tubulogenesis in the intestine. *Dev. Biol.* 272, 262-276. 10.1016/j.ydbio.2004.05.012 15242805

[DEV186817C72] Vastenhouw N. L., Cao W. X. and Lipshitz H. D. (2019). The maternal-to-zygotic transition revisited. *Development* 146, dev161471 10.1242/dev.161471 31189646

[DEV186817C73] Voronina E., Seydoux G., Sassone-Corsi P. and Nagamori I. (2011). RNA granules in germ cells. *Cold Spring Harb Perspect Biol.* 3, a002774 10.1101/cshperspect.a002774 21768607 PMC3225947

[DEV186817C74] Voronina E., Paix A. and Seydoux G. (2012). The P granule component PGL-1 promotes the localization and silencing activity of the PUF protein FBF-2 in germline stem cells. *Development* 139, 3732-3740. 10.1242/dev.083980 22991439 PMC3445306

[DEV186817C75] Walker A. K., Boag P. R. and Blackwell T. K. (2007). Transcription reactivation steps stimulated by oocyte maturation in C. elegans. *Dev. Biol.* 304, 382-393. 10.1016/j.ydbio.2006.12.039 17291483 PMC1913287

[DEV186817C76] Wan G., Fields B. D., Spracklin G., Shukla A., Phillips C. M. and Kennedy S. (2018). Spatiotemporal regulation of liquid-like condensates in epigenetic inheritance. *Nature* 557, 679-683. 10.1038/s41586-018-0132-0 29769721 PMC6479227

[DEV186817C77] Wang J. T., Smith J., Chen B. C., Schmidt H., Rasoloson D., Paix A., Lambrus B. G., Calidas D., Betzig E. and Seydoux G. (2014). Regulation of RNA granule dynamics by phosphorylation of serine-rich, intrinsically disordered proteins in C. elegans. *Elife* 3, 1-23. 10.7554/eLife.04591 PMC4296509 25535836

[DEV186817C78] Zevian S. C. and Yanowitz J. L. (2014). Methodological considerations for heat shock of the nematode Caenorhabditis elegans. *Methods* 68, 450-457. 10.1016/j.ymeth.2014.04.015 24780523 PMC4112136

[DEV186817C79] Zhang Y., Foster J. M., Kumar S., Fougere M. and Carlow C. K. S. (2004). Cofactor-independent phosphoglycerate mutase has an essential role in Caenorhabditis elegans and is conserved in parasitic nematodes. *J. Biol. Chem.* 279, 37185-37190. 10.1074/jbc.M405877200 15234973

[DEV186817C80] Zhang Y., Foster J. M., Nelson L. S., Ma D. and Carlow C. K. S. (2005). The chitin synthase genes chs-1 and chs-2 are essential for C. elegans development and responsible for chitin deposition in the eggshell and pharynx, respectively. *Dev. Biol.* 285, 330-339. 10.1016/j.ydbio.2005.06.037 16098962

