# Supplementary material for: mRNA localization is linked to translation regulation in the Caenorhabditis elegans germ lineage
Source: Development. 2020 Jul 8;147(13):dev186817. doi: 10.1242/dev.186817 (PMC7358130; doi:10.1242/dev.186817)
Supplement: Supplementary information [file develop-147-186817-s1.pdf]

Figure S1

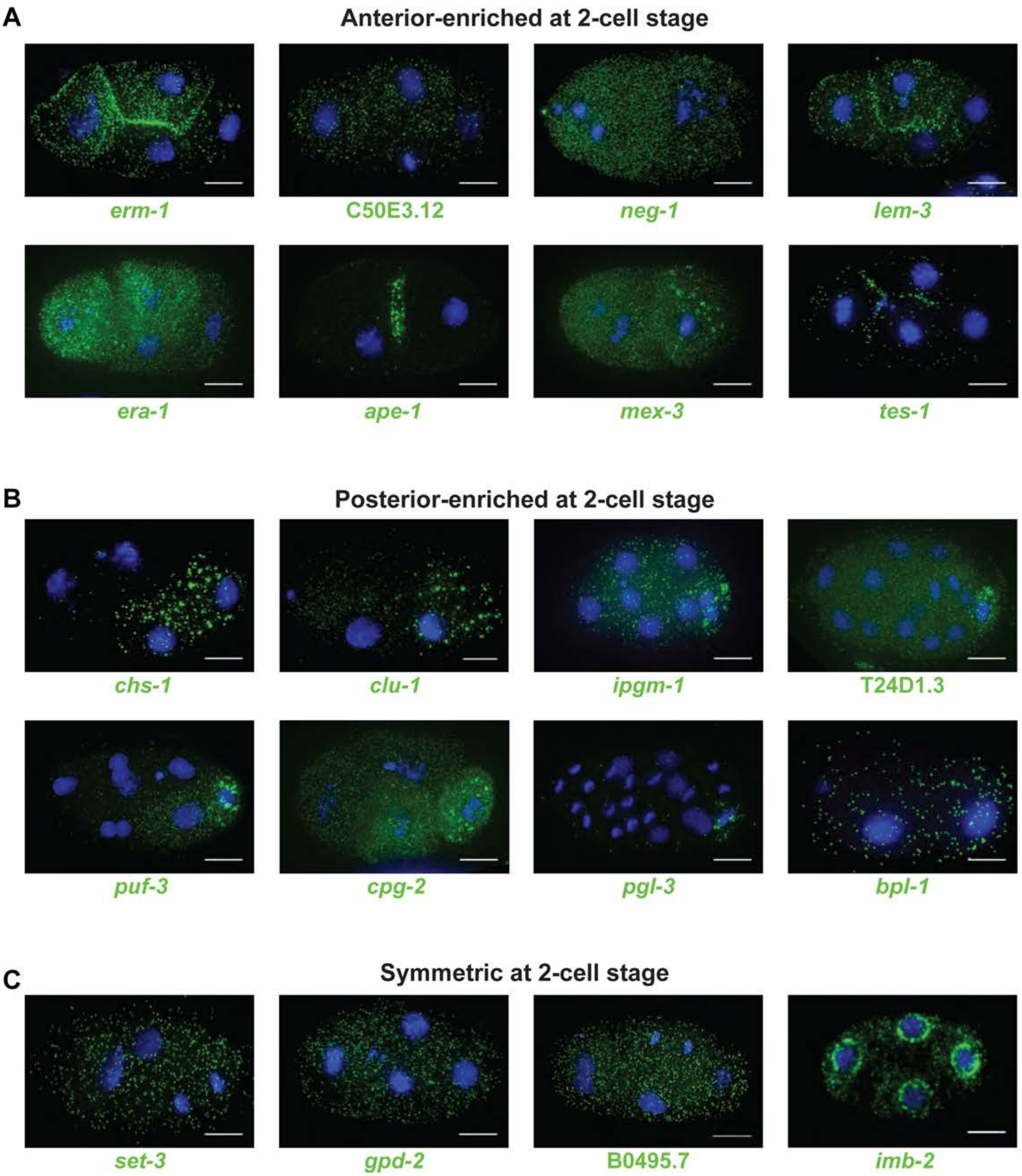
A
Anterior-enriched at 2-cell stage
erm-1
C50E3.12
neg-1
lem-3
era-1
ape-1
mex-3
tes-1
B
Posterior-enriched at 2-cell stage
chs-1
clu-1
ipgm-1
T24D1.3
puf-3
cpg-2
pgl-3
bpl-1
C
Symmetric at 2-cell stage
set-3
gpd-2
B0495.7
imb-2

Fig. S1. Subcellular localization patterns of surveyed transcripts. 8 AB-enriched, 8 P_1-enriched, and 4 symmetric transcripts were selected for verification and examination by smFISH with all transcripts in green and DAPI in blue. **(A)** AB-enriched transcripts *erm-1 (Ezrin/Radixi/Moesin),* C50E3.12, *neg-1 (Negative Effect on Gut development, lem-3 (LEM domain protein), era-1 (Embryonic mRna Anterior)*, *ape-1 (APoptosis Enhancer), mex-3 (Muscle EXcess),* and *tes-1(TEStin homolog)* are shown. **(B)** P_1-enriched transcripts *chs-1 (CHitin Synthase), clu-1 (yeast CLU related), ipgm-1 (cofactor Independent PhosphoGlycerate Mutase homolog),* T24D1.3, *puf-3 (PUF domain containing), cpg-2 (Chondroitin ProteoGlycan), pgl-3 (P-GranuLe abnormality),* and *bpl-1 (Biotin Protein Ligase)* are shown. **(C)** Uniformly distributed transcripts *set-3 (SET domain containing), gpd-2 (Glyceraldehyde 3-Phosphate Dehydrogenase),* B0495.7, *imb-2 (IMportin Beta family)* are shown. Tabulation of the results are in Table 1.

Development • Supplementary information

Figure S2

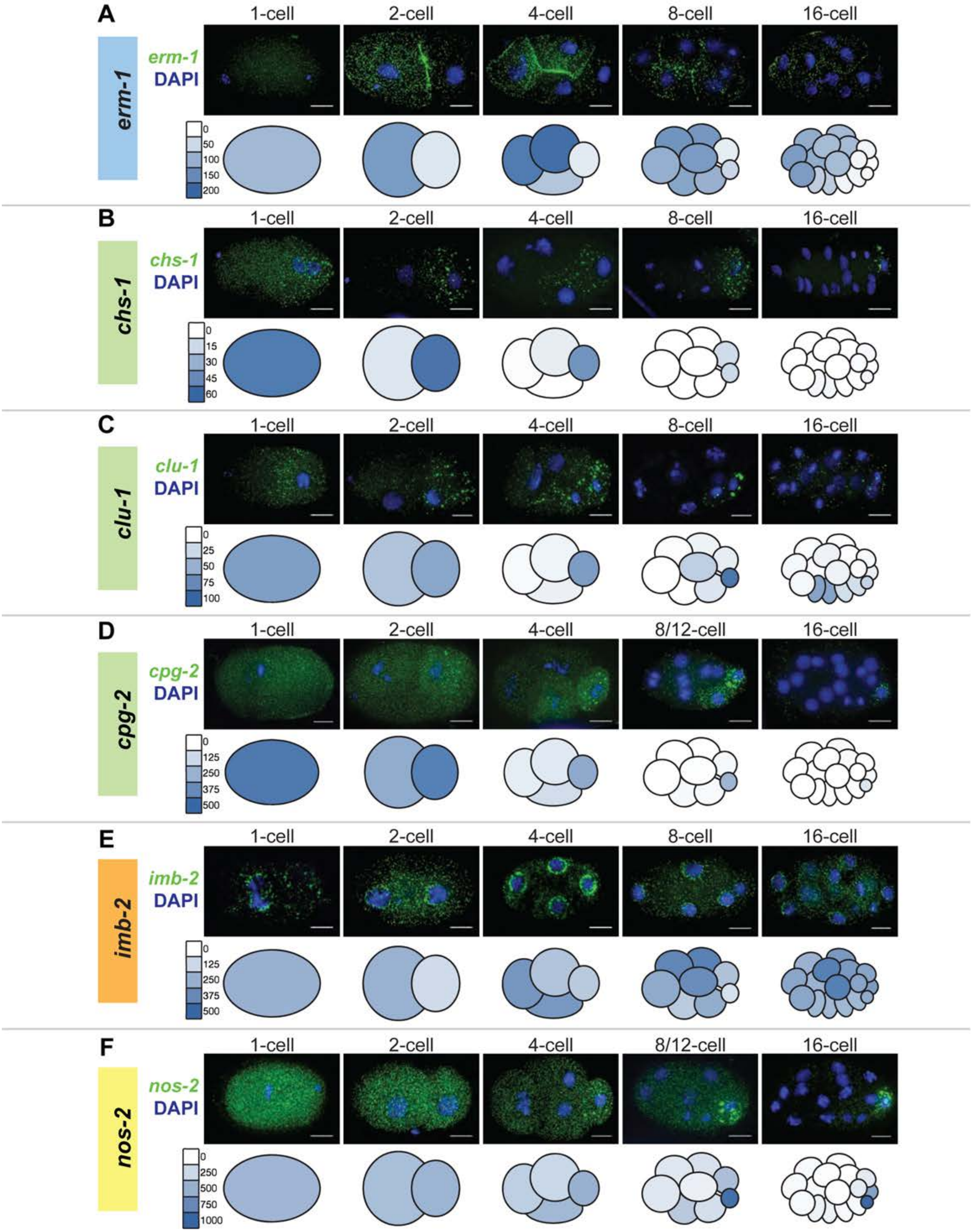

Development • Supplementary information

Fig. S2. Localization patterns of mRNA over developmental time. smFISH microscopy localizations of *erm-1* **(A)**, *chs-1* **(B)**, *clu-1* **(C)**, *cpg-2* **(D)**, *imb-2* **(E)**, and *nos-2* **(F)** shown from 1-cell stage zygotes to the 16-stage. mRNA signal is in green. DAPI is in blue. Below each series of images is single-cell RNA-seq data from the same transcript (from Tintori et al. 2016).

Development • Supplementary information

Figure S3

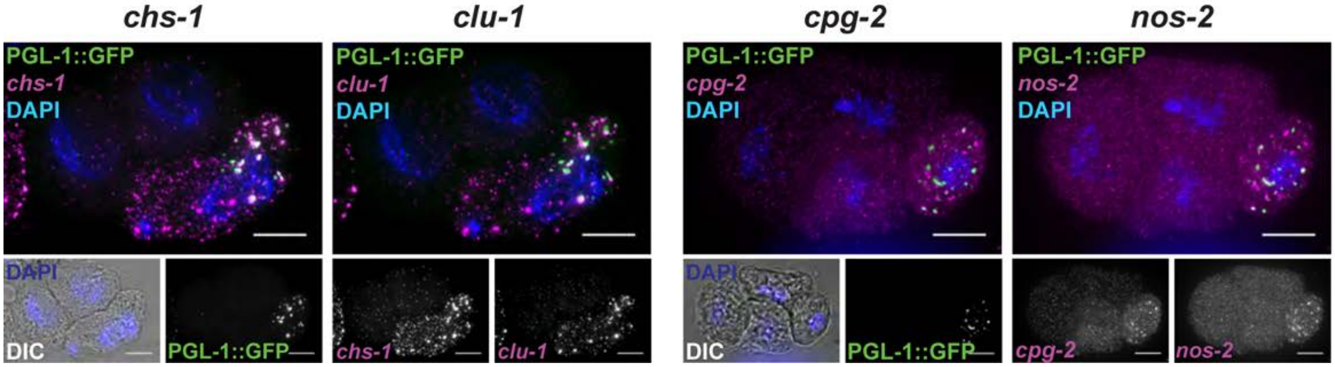

Fig. S3. P_1-enriched, clustered transcripts co-localize with the P granule marker PGL-1::GFP. In addition to co-localizing with GFP signal in the P granule marker strain containing GLH-1::GFP (Fig. 3), *chs-1*, *clu-1*, *cpg-2*, and *nos-2* mRNAs (all in magenta) also co-localize with a second P granule marker protein, PGL-1::GFP (green). DAPI is illustrated in blue to visualize nuclei and illustrate the 4-cell stage of development.

Figure S4

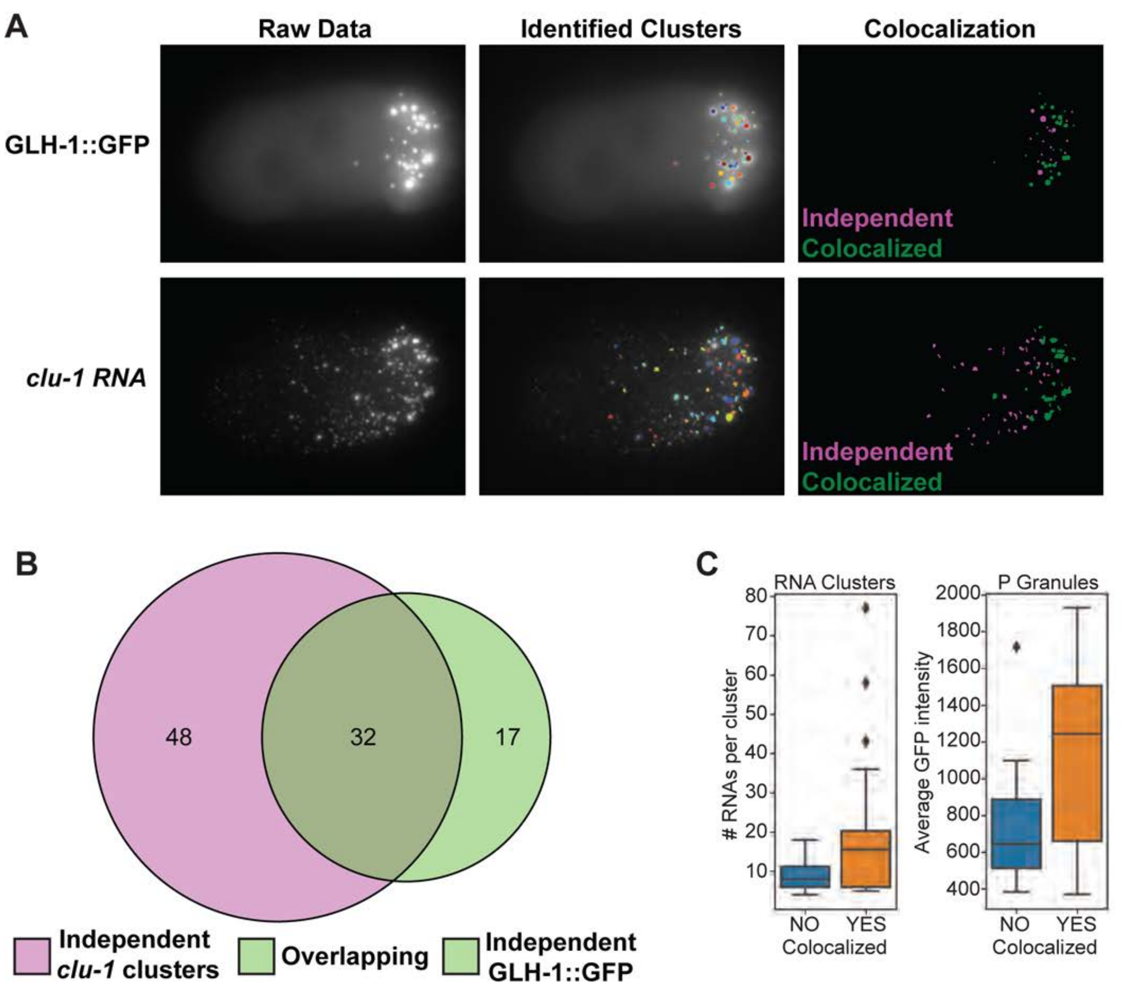

Fig. S4. Quantification of mRNA cluster overlap with the P granules. mRNA cluster overlap with GLH-1::GFP labeled P granules is calculated using micrographs of GLH-1::GFP and clustered RNAs (*clu-1* shown) **(A, left)**, computationally identifying P granules and RNA clusters **(A, middle)**, and comparing the 3D masks for overlap to identify independent P granules and RNA clusters (magenta) or colocalized clusters (green) **(A, right)**. **(B)** A Venn-Euler diagram illustrating the number of independent *clu-1* mRNA clusters (magenta), independent P granules (light green), and overlapping P granules and mRNA clusters (dark green) in a single embryo (from A). **(C)** Box plots comparing the size of non-overlapping mRNA clusters and P granules to those overlapping shows larger mRNA clusters more commonly overlap with P granules (left) and brighter P granules more commonly overlap with mRNA clusters (right).

Figure S5

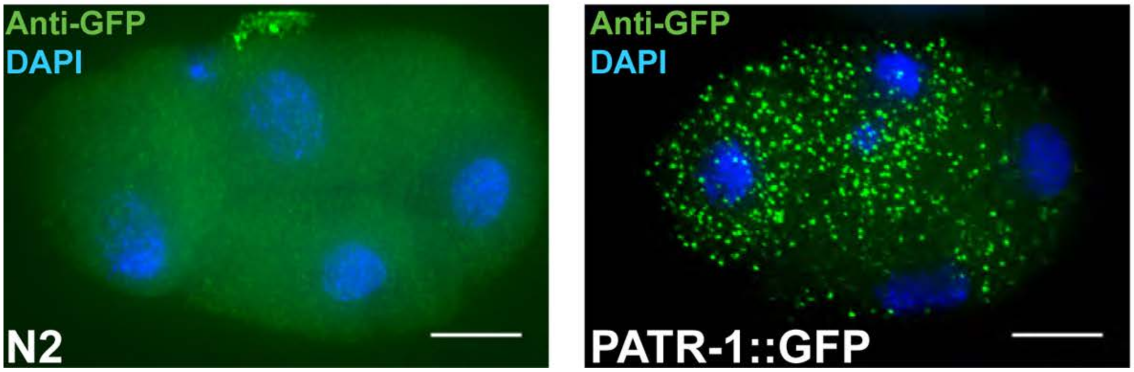

Fig. S5. Immunofluorescence control images for anti-GFP antibody staining. The anti-GFP antibody reports PATR-1::GFP localization **(green, right)** in PATR-1::GFP containing strains as compared to N2 wild type un-transformed control strains **(green, left)**. DAPI staining is shown in blue to visualize nuclei and illustrate the 4-cell stage of development.

Figure S6

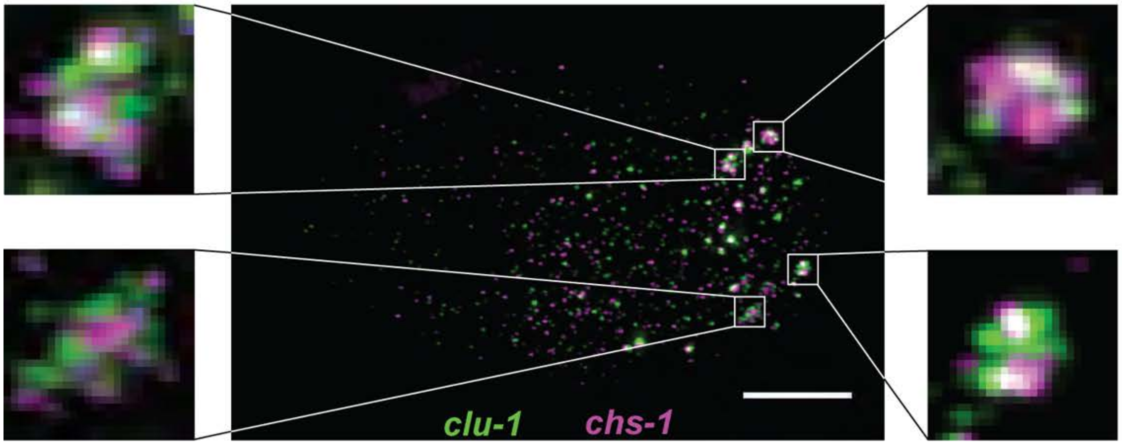

Fig. S6. mRNA clusters display homotypic clustering within P granules. *chs-1* mRNA (magenta) tend to homotypically cluster in the core of P granules while *clu-1* mRNA (green) also cluster homotypically, but near the peripheries of P granules.

Development • Supplementary information

Figure S7

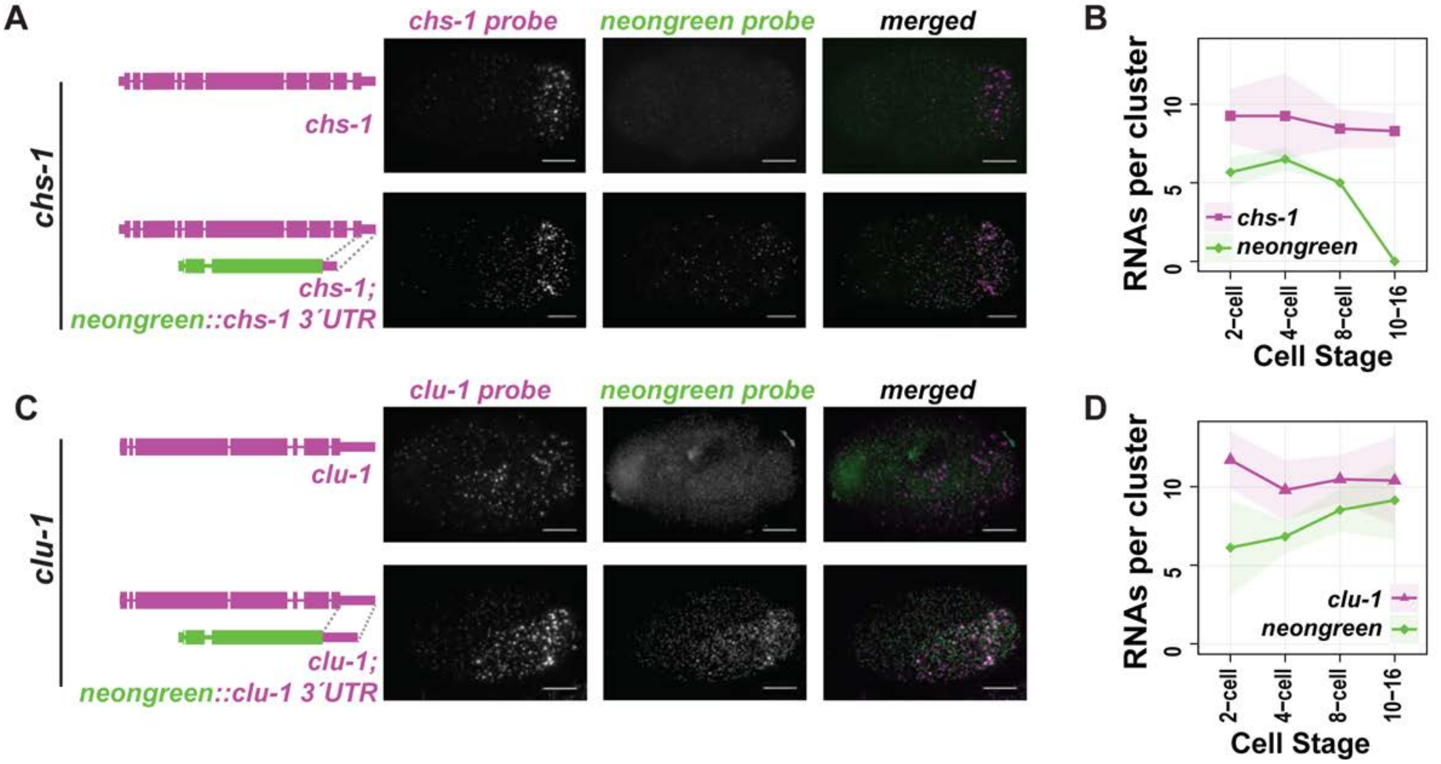

Fig. S7. *chs-1* and *clu-1* 3´UTRs direct neongreen mRNAs to subcellular clusters. **(A)** A single copy of the ***chs-1*** 3´UTR appended to monomeric NeonGreen (*mex-5p::mNeonGreen::chs-1 3´UTR*) was transgenically introduced into otherwise wild-type worms to test whether the 3´UTR of *chs-1* was sufficient to drive the cell peripheral localization pattern observed for full length *chs-1* mRNA. Wild-type (above) and transgenic (below) 4-cell stage embryos were imaged by smFISH using probes to the endogenous *chs-1* mRNA (left), mNeonGreen mRNA (middle), and the channels were then merged (right). **(B)** Quantification of (A) over developmental time. **(C)** As in (A) with using the *clu-1* 3´UTR sequence. (D) As in (B) using the *clu-1* 3´UTR. A minimum of n = 5 for each stage and transcript were collected over 3 biological replicates in A-D. p-values from multiple test corrected t-tests are shown (NS > 0.05; 0.05 > * > 0.005)

Development • Supplementary information

Figure S8

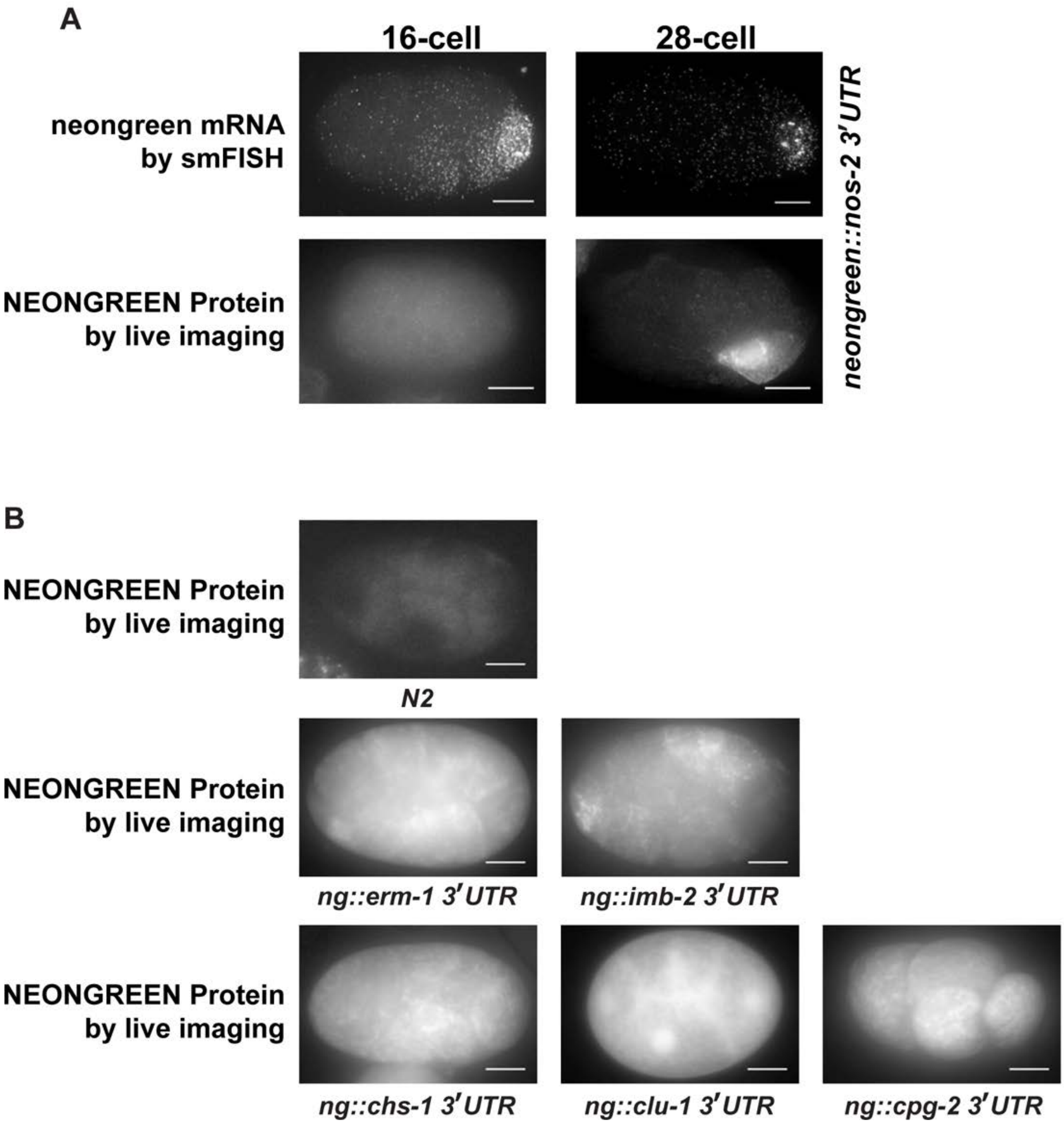

Fig. S8. *Pmex-5::mNeonGreen::nos-2 3´UTR* RNA recapitulates endogenous translation repression and activation. (A, left) A *Pmex-5::mNeonGreen::nos-2 3´UTR* embryo at the 16-cell stage. smFISH for mNeonGreen RNA demonstrated normal RNA localization. Epifluorescent microscopy of living *Pmex-5::mNeonGreen::nos-2 3´UTR* embryos at the 16-cell stage showed no expression of the mNeon-Green reporter protein. **(A, right)** As in (A) at the 28-cell stage. Epifluorescent microscopy of living *Pmex-5::mNeonGreen::nos-2 3´UTR* embryos at the 28-cell stage showed P lineage specific expression of the mNeonGreen reporter protein. **(B)** Epifluorescent microscopy of wildtype N2 embryos demonstrates no apparent fluorescence while *Pmex-5::mNeonGreen::3´UTR* of Interest embryos show low levels of cell-non-specific mNeonGreen fluorescence.

Figure S9

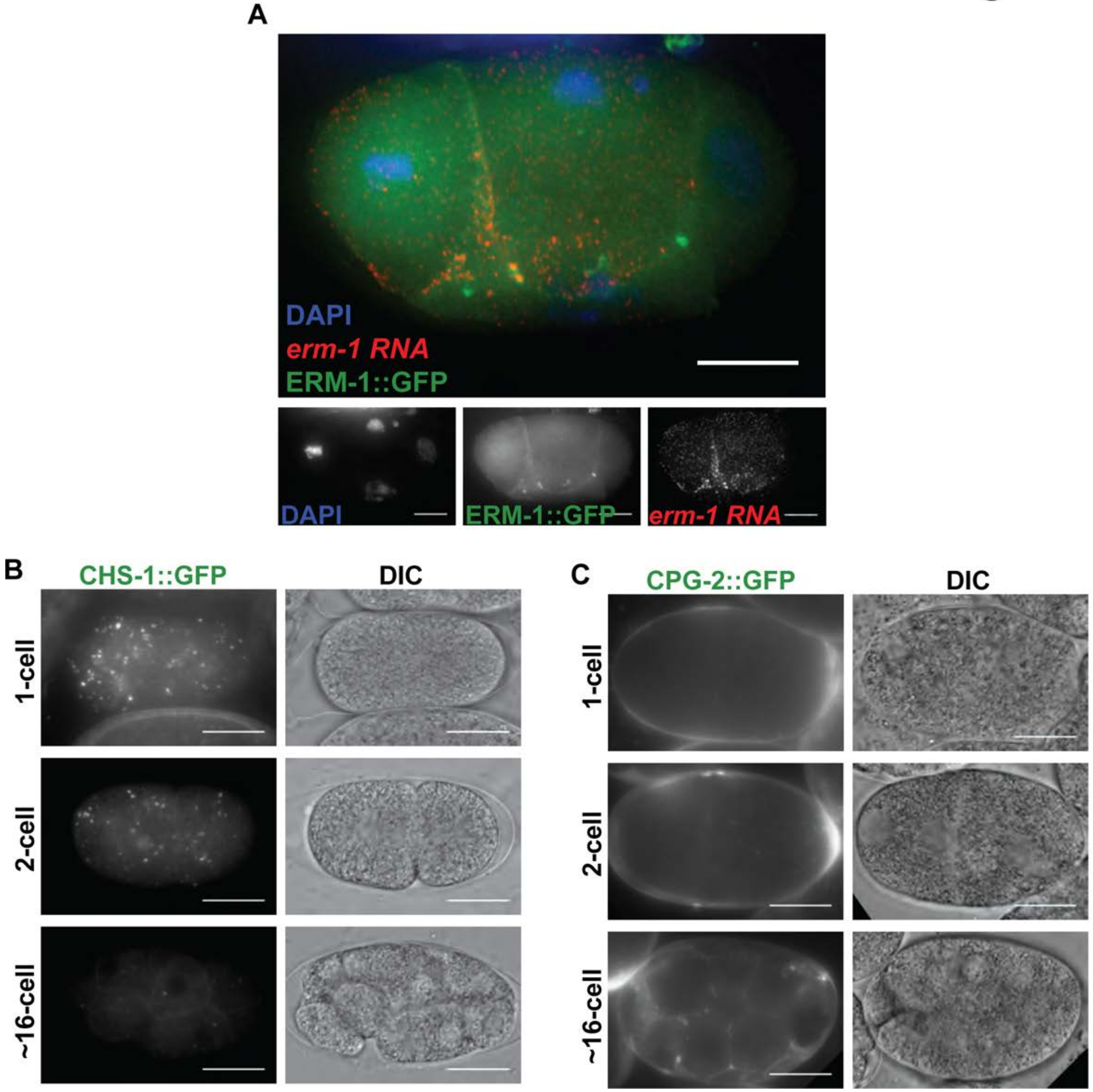

Fig. S9. *erm-1::GFP* RNA localizes like endogenous *erm-1*. (A) smFISH microscopy of *Perm-1::erm-1 ORF::GFP::erm-1 3′UTR* RNA (green) colocalizes with endogenous *erm-1* RNA (red). **(B)** Epifluorescent microscopy of CHS-1::GFP embryos at the 1, 2, and 16-cell stages of embryogenesis show gradual depletion of the CHS-1::GFP protein puncta. **(C)** As in (B) imaging CPG-2::GFP embryos. CPG-2::GFP protein can be seen in the extracellular space of the embryo, but not within cells.

Development • Supplementary information

Figure S10

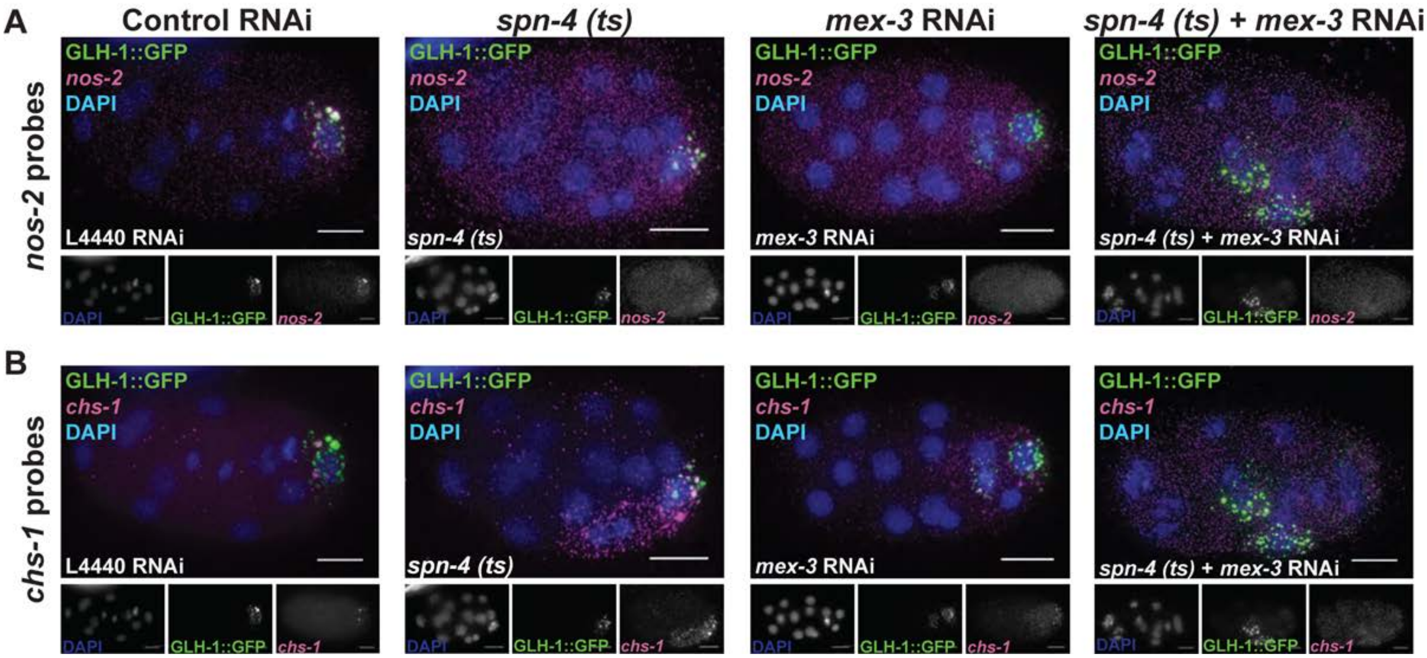

Fig. S10. Knockdown of *spn-4* and *mex-3* simultaneously resulted in embryonic defects and mislocalization of RNA. GLH-1::GFP and GLH-1::GFP *spn-4(or191)* temperature sensitive embryos raised at 25 °C (GLH-1::GFP shown in green) were treated with L4440 or *mex-3 RNAi* and probed for *chs-1* **(A)** and *nos-2* **(B)** mRNAs by smFISH (magenta). DNA was also stained with DAPI (blue). GLH-1::GFP *spn-4(or191)* embryos raised at 15 °C and treated with either L4440 or mex-3 RNAi phenocopied GLH-1::GFP embryos raised at 25 °C with the same RNAi treatment.

Figure S11

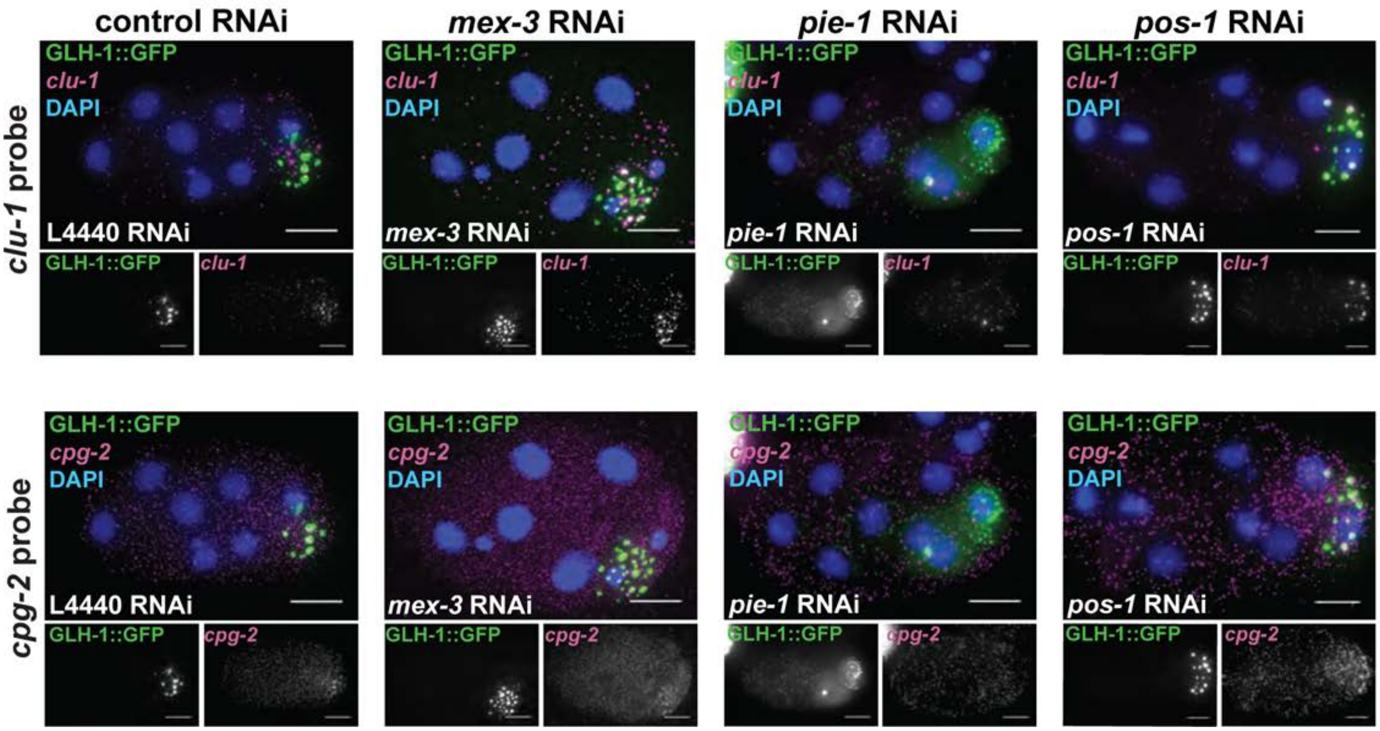

Fig. S11. Knockdown of RBPs important for *nos-2* transcript localization results in perturbed *cpg-2* and *clu-1* transcript localization. GLH-1::GFP (green) embryos were treated with L4440, *mex-3*, *pie-1*, or *pos-1* RNAi and probed for *clu-1* **(A)** and *cpg-2* **(B)** mRNAs by smFISH (magenta). DNA was also stained with DAPI (blue).

Figure S12

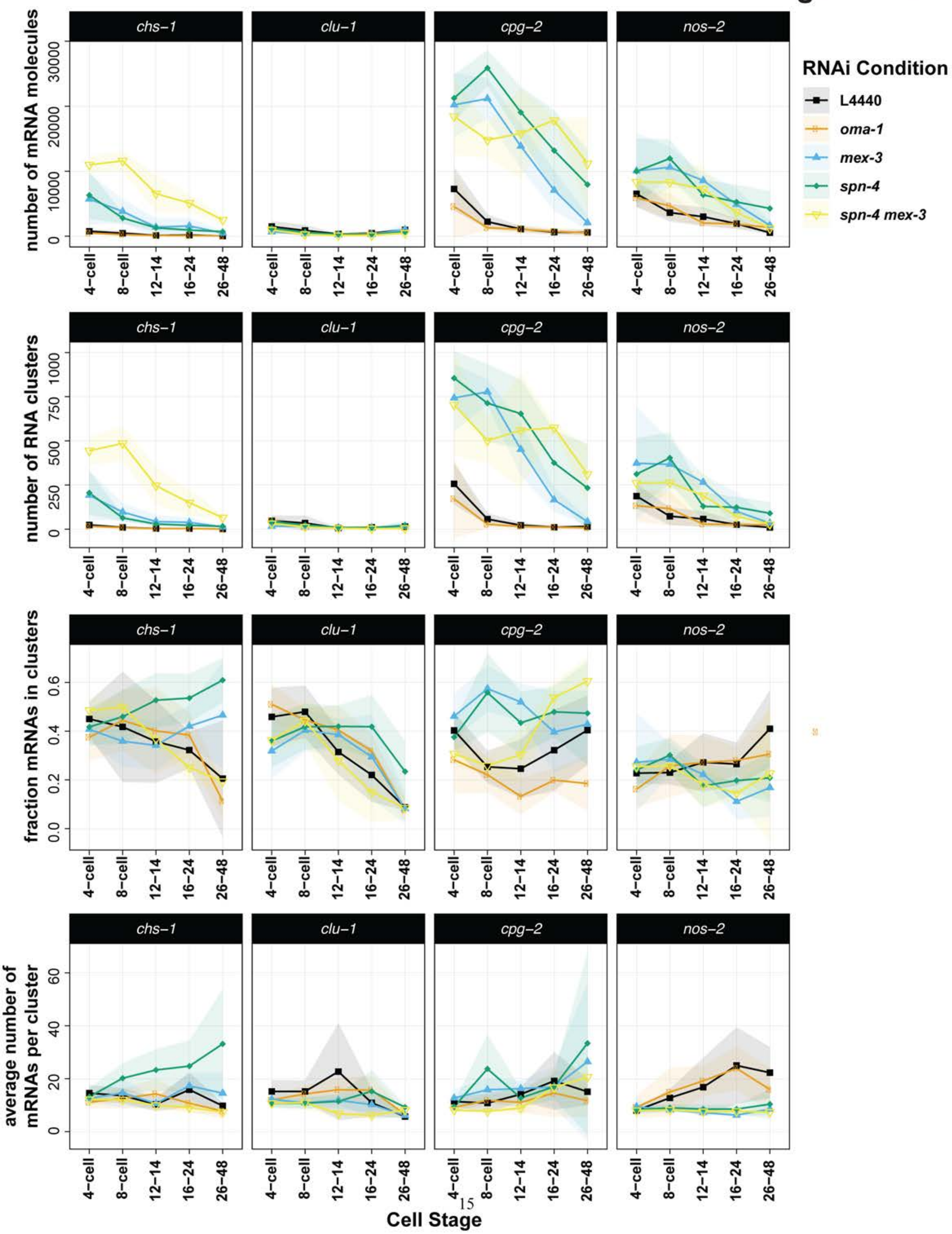

Development • Supplementary information

Fig. S12. Quantification of mRNA clustering under RBP knockdown conditions. Statistical metrics characterizing mRNA clustering are shown: 1) the total number of RNAs in each embryo, 2) the total number of clusters identified in each embryo, 3) the fraction of total mRNAs located within clusters (as opposed to cluster-independent), and 4) the average estimated number of mRNA molecules per cluster within a given embryo. Four transcripts were assayed in five knockdown conditions over 5 developmental time points. The average of each metric (line) and their standard deviation (shading) are shown representing a minimum of 5 embryos assayed over a minimum of 3 biological replicates.

Development • Supplementary information

Figure S13

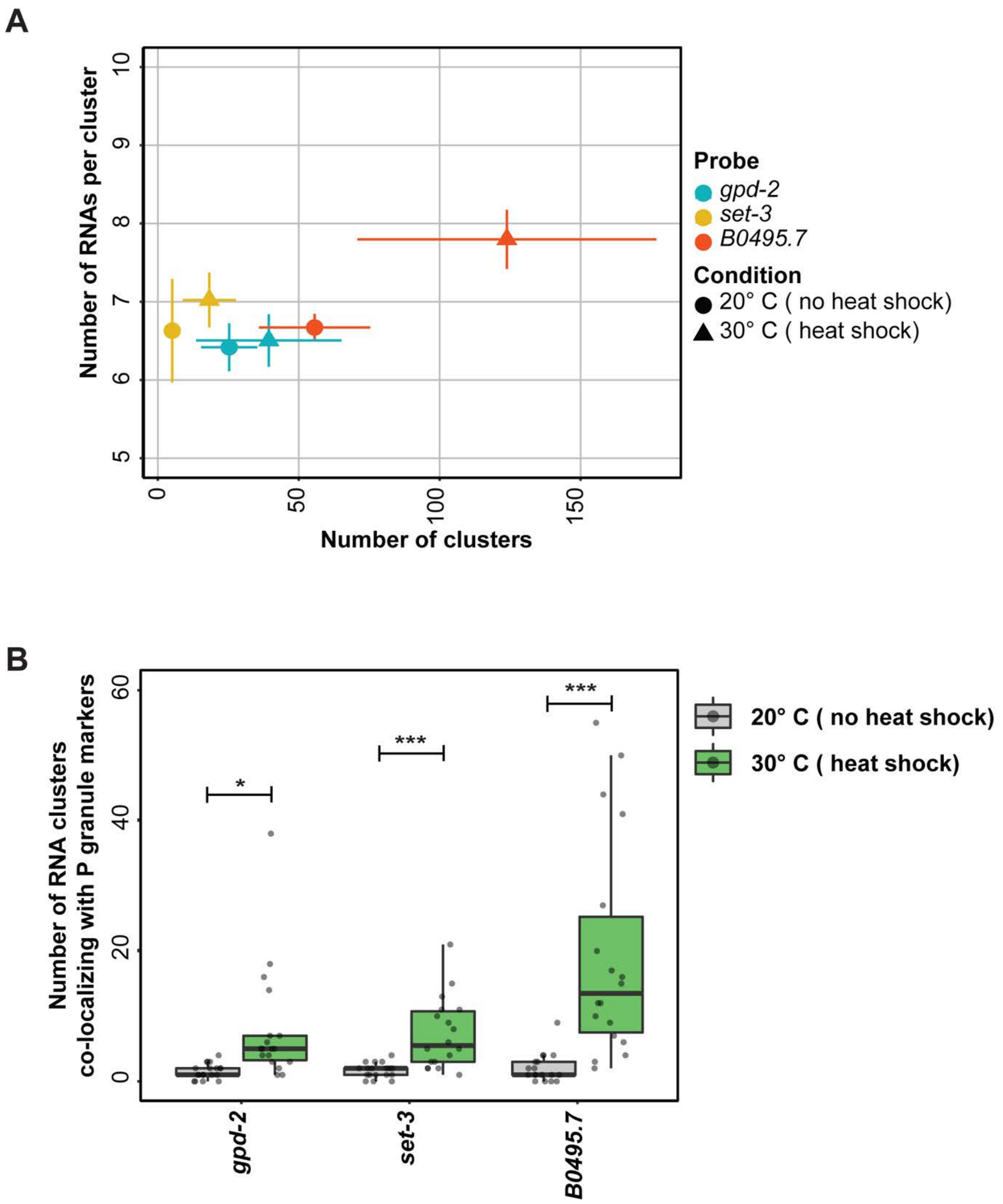

Development • Supplementary information

Fig. S13. Upon heat shock, a greater number of mRNAs clusters co-localize with P granules. Quantification of smFISH assays of *B0495.7*, *gpd-2*, and *set-3*, three transcripts that are typically not P granule localized. **(A)** Upon heat shock (30º C), larger and more numerous clusters formed. **(B)** Upon heat shock, more numerous RNA clusters co-localized with the P granule marker protein (GLH-1::GFP). The percentages of clusters overlapping P granule markers are shown in Figure 7B. Here, the raw numbers of co-localized clusters are tabulated. Statistics were performed using Welch's Two Sample t-test p-values: 0.05 > * ≥ 0.005; 0.005 > ** ≥ 0.0005; *** < 0.0005.

Table S1. Worm strains used in this study.

Click here to Download Table S1

Table S2. *E.coli* strains and plasmids used in this study.

Click here to Download Table S2

Table S3. Oligonucleotide primers used in this study.

Click here to Download Table S3

Table S4. smFISH and smiFISH probesets used in this study.

Click here to Download Table S4

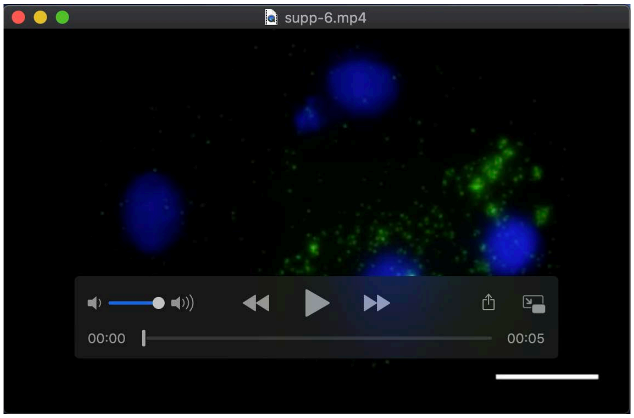

Movie 1. *erm-1* mRNA projection. 3D rendering of stacked images of *erm-1* mRNA as imaged using smFISH in a 4-cell stage embryo.

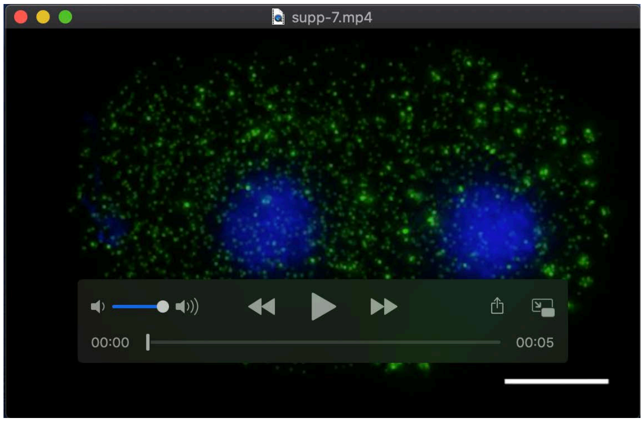

Movie 2. *chs-1* mRNA projection. 3D rendering of stacked images of *chs-1* mRNA as imaged using smFISH in a 4-cell stage embryo.

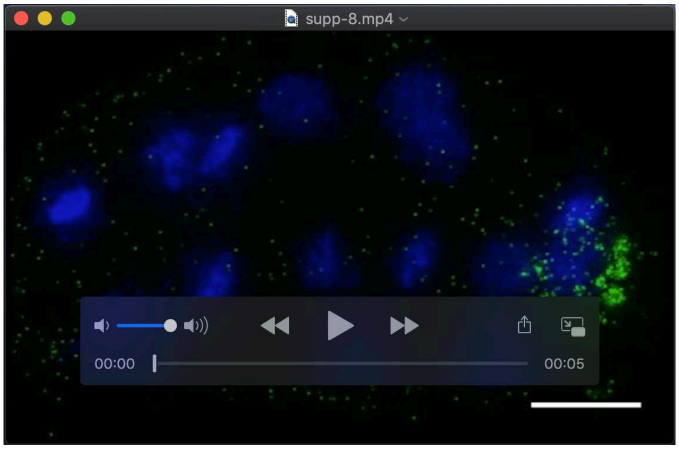

Movie 3. *clu-1* mRNA projection. 3D rendering of stacked images of *clu-1* mRNA as imaged using smFISH in a 4-cell stage embryo.

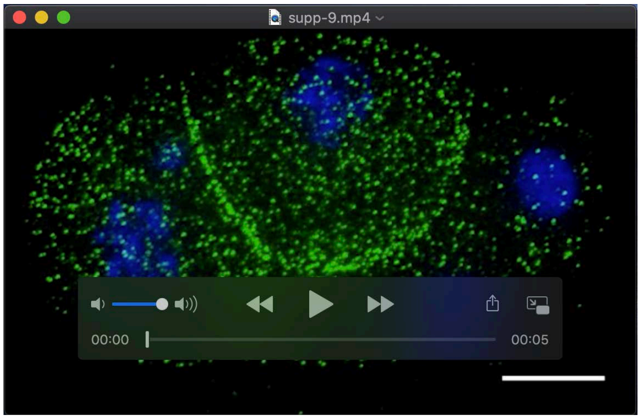

Movie 4. *cpg-2* mRNA projection. 3D rendering of stacked images of *cpg-2* mRNA as imaged using smFISH in a 4-cell stage embryo.

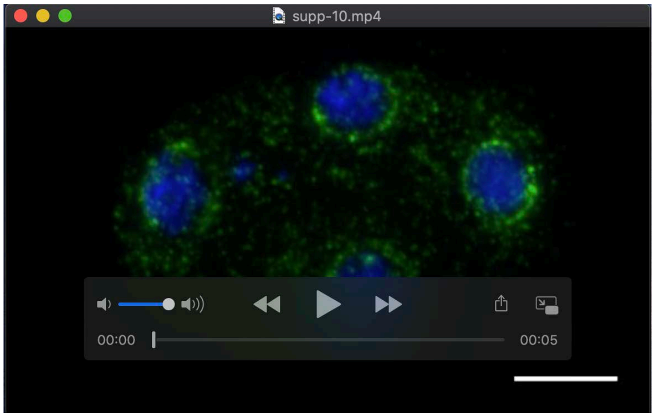

Movie 5. *imb-2* mRNA projection. 3D rendering of stacked images of *imb-2* mRNA as imaged using smFISH in a 4-cell stage embryo.

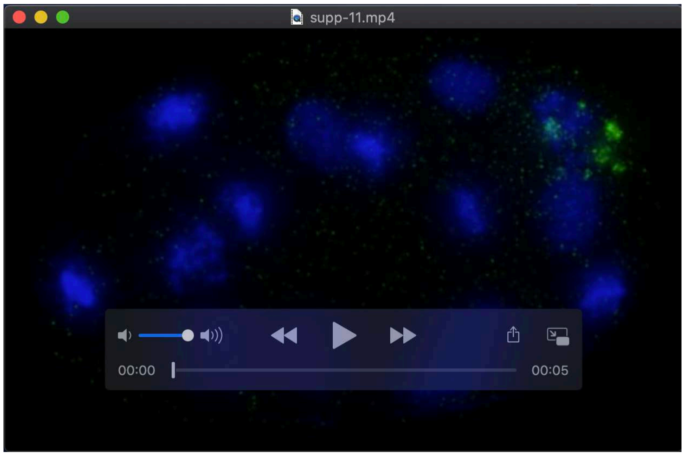

Movie 6. *nos-2* mRNA projection. 3D rendering of stacked images of *nos-2* mRNA as imaged using smFISH in a 4-cell stage embryo.
