# Supplementary material for: mRNA localization is linked to translation regulation in the Caenorhabditis elegans germ lineage
Source: Development. 2020 Jul 8;147(13):dev186817. doi: 10.1242/dev.186817 (PMC7358130; doi:10.1242/dev.186817)
Supplement: Reviewer comments [file develop-147-186817_review_history.pdf]

mRNA localization is linked to translation regulation in the *Caenorhabditis elegans* germ lineage

Dylan M. Parker, Lindsay P. Winkenbach, Samuel Boyson, Matthew N. Saxton, Camryn Daidone, Zainab A. Al-Mazaydeh, Marc T. Nishimura, Florian Mueller and Erin Osborne Nishimura
DOI: 10.1242/dev.186817

Editor: Susan Strome

Review timeline
Original submission: 27 November 2019
Editorial decision: 2 March 2020
First revision received: 14 April 2020
Editorial decision: 4 May 2020
Second revision received: 26 May 2020
Accepted: 30 May 2020

Original submission

First decision letter

MS ID#: DEVELOP/2019/186817

MS TITLE: mRNA localization is linked to translation regulation in the Caenorhabditis elegans germ lineage

AUTHORS: Dylan M. Parker, Lindsay P. Winkenbach, Samuel Boyson, Matthew N. Saxton, Camryn Daidone, Zainab A. Al-Mazaydeh, Marc T. Nishimura, Florian Mueller, and Erin Osborne Nishimura

My sincere apologies for the long review process for your submission, and thanks for your patience. Reviewer 2 never came through, so I stepped in to be Reviewer 2. Our reviewer comments are appended below, or you can access them online: please go to BenchPress and click on the 'Manuscripts with Decisions' queue in the Author Area.

Both Reviewer 1 and I agree that your manuscript presents careful and important analyses and findings that will be of interest to the broad readership of Development. We recommend numerous textual edits that will improve the readability of your paper. I pointed out several figures in which the small bottom panels are so miniature that they don't effectively show the main point. And I questioned some of your p-values. Since the Lee et al. eLife paper from the Seydoux lab is now out, I recommend that you compare and contrast your and their results where it would enhance your paper.

I invite you to consider our suggestions and submit a revised manuscript. Your revised manuscript will be re-reviewed, and acceptance will depend on your satisfactorily addressing the reviewers'concerns. Please note that Development normally permits only one round of 'major revision'.

In your revised manuscript, please clearly HIGHLIGHT all changes made in the revised version. You should avoid using 'Tracked Changes' in Word files as these are lost in PDF conversion. I also request a point-by-point response detailing how you have dealt with the points raised by the reviewers in

© 2020. Published by The Company of Biologists under the terms of the Creative Commons Attribution License (https://creativecommons.org/licenses/by/4.0/).

the ‘Response to Reviewers’ box. If you do not agree with any of the reviewers’ criticisms or suggestions, please explain why.

Reviewer 1

Advance summary and potential significance to field

In recent years, the Osborne-Nishimura lab investigated the asymmetric segregation of maternally loaded transcripts during C. elegans embryogenesis. In this report, the validation of these asymmetries using single-mRNA FISH revealed an added level of post-transcriptional regulation by showing that many transcripts cluster and localize to distinct subcellular regions. These distributions are thoroughly demonstrated in both supplemental and in-text figures. Some findings are very descriptive meaning that no functions are attributed to this localization; however there are limited examples of subcellular transcript distributions or research looking into this phenomenon, so the extensive descriptions here will lay the groundwork for subsequent studies.

The authors find variations on whether 3Â’UTRs direct subcellular distribution, highlighting the potential for diverse regulatory mechanisms.
Next, they focus on the role that translational inhibition plays in the localization of transcripts to maternally inherited P granules in germline blastomeres. They discover that translational inhibition directs transcripts to P granules, possibly to direct primordial germ cell development.

The conclusions presented here are novel and will be of general interest to developmental, RNA, and germ-granule biologists. The report is timely, in that it largely agrees with some findings in a contemporary study coming out soon from the Seydoux lab. Only minor comments follow:

Comments for the author

Line 53. The sentence needs clarification to know what the terminal “it”
refers.
Line 195. The authors note that zygotic transcripts do not have subcellular localization patterns. Could part of this be due to smaller cell sizes in the embryo upon zygotic transcription?
Line 246. This sentence is difficult to read. Restate to state that some signals come from more than just one transcript – or something.
Line 357. More informative than what? What is concluded from the results in this paragraph?
Line 371. show(s)
Line 402. Clarify what is being rectified.
The paragraph on page 15, especially lines 450-459, is very hard to read.
Consider a simple figure to help readers get through the reasoning.
Line 448. A reference should be included for the somatic gene expression that coincides with PIE-1 disruption.
Line 466. The statement is made that nos-2 remains translationally repressed despite no P-granule localization. What is the evidence for this translational repression?
Line 560. specie(s)
Line 612. Correlates
Line 613. This sentence saying that mRNA correlates with post-
transcriptional control is redundant. mRNA is already post-transcribed by definition. Clarify.

First revision

Author response to reviewers' comments

April 3, 2020

We are pleased to submit our revised research article “mRNA localization is linked to translation regulation in the *Caenorhabditis elegans* germ lineage” for your consideration.

© 2020. Published by The Company of Biologists under the terms of the Creative Commons Attribution License (https://creativecommons.org/licenses/by/4.0/).

In our previous manuscript, we presented our discovery of maternally inherited mRNA transcripts that accumulate at subcellular locations in early embryos localizing to cell membranes, nuclear membranes, P granules, and P bodies. We worked to preliminarily characterize cis- and trans-factors involved in the localization of these transcripts. In doing so, we found that membrane-associated mRNAs were linked to translational activity and local protein accumulation whereas P granule associated mRNAs were linked to either temporary or permanent translational repression. In further studying the localization of *nos-2* to P granules, we were able to answer a long-standing curiosity in the field… are mRNAs recruited to P granules for the purpose of translational repression or does translational repression itself drive mRNAs into P granules? In the case of *nos-2,* we found three lines of evidence supporting the second model: 1) P granule localization occurs after translational repression, 2) translational repression does not depend on P granule localization, and 3) translational disruption is sufficient to direct transcripts to P granules.

In response to the reviewer's insightful comments, we amended our manuscript in the following ways that greatly improved its accuracy, insight, and readability:

- **Figure 2B.** We discovered that some of the control data for Figure 2B was repeated from another figure. We have rectified this and substituted the independent dataset.

- **Figure 3.** We increased the size of some panels in Figure 3, as per reviewer #2's recommendations, with improved visibility.

- **P value.** We took the reviewer #2's suggestion to simplify the way that we demarcated adjusted p values in Figure 2 and Figure 5.

- **Figure reference notation.** We modified the figure referencing within the manuscript to comply with the Development format.

- **Addressed the issue of shrinking cell sizes.** Reviewer #1 raised the astute point that it may be difficult to assess localization of zygotically expressed transcripts given the shrinking size of each cell. We amended the text by acknowledging this possibility and explained that the genes we have selected are present at or prior to the 16-cell stage when localization is still discernable for most patterns.

- **Discussion.** We restructured the discussion. The new discussion incorporates new findings from a recently published study (Lee et al., 2020, eLife: doi://10.7554/eLife.52896). The new discussion is shorter in compliance with manuscript size recommendations.

- **Manuscript size.** We edited the manuscript down by removing excess wording, largely from the introduction and discussion.

- **References.** We added several references recommended by both Reviewer #1 and Reviewer #2, and we moved some references within the text to improve clarity.

- **Full dataset repository.** We deposited the full compendium of all raw microscopy images with the Colorado State University Library and have included a link to that repository. This is part of the CSU's increased effort for best practices in data archiving and reproducible research.

- **Github.** In places where the analysis has been updated (Figure 2B), we updated our GitHub repository to reflect the updates.

- **Grammar.** We fixed many grammatical issues both reviewers thankfully identified.

- **Point-by-point.** A point-by-point description of all changes that address points made by both reviewers accompanies this letter.

The changes we implement at the suggestion of the two reviewers have greatly improved the manuscript. We thank the reviewers for their thoughtful consideration of the work. We hope the

© 2020. Published by The Company of Biologists under the terms of the Creative Commons Attribution License (https://creativecommons.org/licenses/by/4.0/).

reviewers will share our opinion that the manuscript is improved and can deem it acceptable for publication in Development.

Addressing general comments:

- Compare results to Lee et al. paper
- Action: We re-wrote the discussion to include this reference and a discussion of their work.

Addressing comments from Reviewer #1:

- Line 53. The sentence needs clarification to know what the terminal "it" refers.
- Comment: Line 53 is blank space in the document I'm looking at. Does this refer to the terminal "it" in line 44?
- Action: Line 44: Replaced terminal "it" with "repression."

- Line 195. The authors note that zygotic transcripts do not have subcellular localization patterns. Could part of this be due to smaller cell sizes in the embryo upon zygotic transcription?
- Comment: This is an issue in mid- to late-embryogenesis, but the zygotic transcripts we tested were expressed in the 4-cell to 16-cell stage embryos where cytoplasmic patterning, if present, would still be clear.
- Action: added "In mid to late embryonic stages, the size of each cell shrinks, obscuring observations of mRNA patterning. However, the zygotic transcripts we tested were visible at or prior to the 16-cell stage, early enough to avoid this issue. Therefore, we were able to determine…"

- Line 246. This sentence is difficult to read. Restate to state that some signals come from more than just one transcript – or something.
- Action: Line 255 (after edits): Replaced the sentence ". To overcome this, we used a tiered approach, first identifying individual mRNAs (Mueller et al., 2013) and secondly applying the fluorescence intensities and volumes of the individuals to fit a Gaussian Mixture Model (GMM) that estimates the number of molecules contributing to signal overlap (see Methods)." With "To overcome the difficulties in quantifying overlapping smFISH signals, we used a tiered approach, first identifying individual mRNAs (Mueller et al., 2013) before estimating the number of molecules contributing to signal overlap by fitting a Gaussaian Mixture Model (GMM) to the average fluorescence intensities and volumes of the individual molecules (see Methods)."

- Line 357. More informative than what? What is concluded from the results in this paragraph?
- Action: Line 371-377 (after edits): Moved final sentence from the first paragraph of this section to be the first sentence of the next paragraph separate different thoughts. Also added some clarifying language to this sentence to lead into the next paragraph better. Additionally, added some clarifying language to the last sentence of the first paragraph to emphasize that the NEONGREEN 3'UTR reporters did not give deep insights into when the localized transcripts are actively translated.

- Line 371. show(s)
- Action: Line 394 (after edits): Changed show to shows.

- Line 402. Clarify what is being rectified.
- Action: Line 426 (after edits): Replaced "due to limitations of previous techniques" with "due to a lack of single-molecule resolution FISH data under knockdown conditions"

- The paragraph on page 15, especially lines 450-459, is very hard to read. Consider a simple figure to help readers get through the reasoning.

© 2020. Published by The Company of Biologists under the terms of the Creative Commons Attribution License (https://creativecommons.org/licenses/by/4.0/).

- Action: We rewrote this paragraph for clarity
- Action: Still need to make a little figure.

- Line 448. A reference should be included for the somatic gene expression that coincides with PIE-1 disruption.
- Action: Line 502 (after edits): Added reference to Seydoux et al. 1996.

- Line 466. The statement is made that nos-2 remains translationally repressed despite no P-granule localization. What is the evidence for this translational repression?
- Action: We added the references in this section that have reported that POS-1 disruption leads to loss of *nos-2* translation (D'Agostino et al., 2006; Jadav et al., 2008) and the references that PIE-1 disruption leads to loss of *nos-2* translation (Tennehaus et al., 2001)

- Line 560. specie(s)
- Action: Line 628 (after edits): Corrected "specie" to "species."

- Line 612. Correlates
- Action: Line 681 (after edits): Corrected "correlating" to "correlates.:

- Line 613. This sentence saying that mRNA correlates with post-transcriptional control is redundant. mRNA is already post-transcribed by definition. Clarify.

o Action: Line 681 (after edits): Added "subcellular localization" to clarify and specify what is correlating with post-transcriptional regulation.

<u>**Addressing comments from Reviewer #2:**</u>

o Line 103: Change to "biases in mRNA location can precede"
o Action: Line 103: Added "localization."

o Line 113: Change "Geraldine Seydoux" to "Seydoux"
o Action: Line 113: Changed "Geraldine Seydoux" to "Seydoux"

o Line 151: Change "more numerous" to "additional"
o Action: Line 155 (after edits): Changed "more numerous" to "additional"

o Line 164: Define smFISH and smiFISH
o Action: Line 168-170 (after edits): Defined smFISH and smiFISH.

o Line 196: "… maternally loaded mRNAs may be over-represented for subcellular localization" is awkward. Perhaps change to "… maternally loaded mRNAs are more likely than zygotically produced mRNAs to be restricted to particular subcellular locations" or something like that. Also see Reviewer 1's good point about this.
o Action: Line 202 (after edits): Took edit verbatim.

- Line 487: Please provide a reference for heat repressing protein synthesis.
- Action: provided

o Table 1 legend: Please explain the "ranking" value in column 3, and the mex-3 row (AB-enriched, granular (in AB?), and the note that granules are in the P lineage).
o Action: Line 939-940 and 943-944: Addressed both ranking and *mex-3* granules.

o Fig 1 legend Line 871: Change "emphasize" to "summarize"
o Action: Line 962 (after edits): Changed "emphasize" to "summarize."

o Fig 2: I am confused. The main text (line 258) and legend both mention five stages, when

© 2020. Published by The Company of Biologists under the terms of the Creative Commons Attribution License (https://creativecommons.org/licenses/by/4.0/).

six stages are shown. What are the p-values relative to? What do the <*5 and *5 p-values mean? It would seem preferable to have 4 asterisks represent all p-values <0.00005.

- Action: Line 298 (after edits): Changed "at five stages" to "at six stages"
- Action: Line 992 (after edits): Changed "at five stages" to "at six stages"
- Action: amended the figure legend to specify that the P values are a comparison between the transcript of interest and the control (un-clustered) transcript *set-3*.
- Action: Changed Figure 2 as Reviewer #2 suggested. 4 asterisks now represent all p values < 0.00005.
- Action: Changed Figure 5 also: 4 asterisks now represent all p values < 0.00005. Also updated Figure 5 legend to specify the critical comparison made

- Fig 3B: The "DAPI" label in blue is hard to see.
- Action: We have lightened the text in this figure and all other figures that include "DAPI" written in that darker color, both main figures & supplemental figures.

- Fig 3C: Is the quantitation from 4-cell embryos?If yes, specify that in the legend and in line 289 move "(Fig. 3C)" to after 4-cell stage; Fig. 3C does not show "increasing thereafter".
- Action: Line 1003: Added "in 4-cell embryos"
- Line 310 (after edits): Moved Fig. 3C to follow 4-cell stage.
- Line 311: Added "(data not shown)" following "increased thereafter"

- Fig 3D: In several figures, the bottom small panels are so miniature that they don't effectively show the main point. Please consider showing regions of the embryos at higher magnification and/or adding arrows or boxes to show the same granule or region in each black and white image.
- Action: We re-sized these images. We added arrows for clarity as suggested.

- Fig 6C: "prolonged" is misspelled
- Action: fixed this error

<u>**Line-by-line changes**</u>

- Line # is from the original document

Line 46: Changed "can be" to "are"
Line 47: "Can direct" to "directs"
Line 48: Added "their" for clarity
Line 113: Changed "Ribonucleoprotein particles (RNPs)" to "phase-separated condensates"
Line 115: Changed "mRNAs associate with P granules for diverse purposes depending on the transcript" to "transcripts, like *nos-2* and others, associate with P granules for the purpose of forcing their translational repression"
Line 175: Changed figure references to include Figure 1 and Figure S1 instead of just figure S1.
Line 175: Changed "Figure" to "Fig."
Line 186: Changed "Figure" to "Fig."
Line 188-189: Changed "Figure" to "Fig."
Line 192: Added "In mid to late embryonic stages, the size of each cell shrinks, obscuring observations of mRNA patterning. However, the zygotic transcripts we tested were visible at or prior to the 16-cell stage, early enough to avoid this issue. Therefore, we were able to determine"
Line 204: Added a link to Figure 2C where quantification demonstrates localization.
Line 206: Changed "Figure" to "Fig."
Line 208: Changed "Figure" to "Fig."
Line 215: Changed "Figure" to "Fig."
Line 217: Changed "Figure" to "Fig."
Line 221: Changed "Figure" to "Fig."
Line 223: Changed "Figure" to "Fig."

© 2020. Published by The Company of Biologists under the terms of the Creative Commons Attribution License (https://creativecommons.org/licenses/by/4.0/).

Line 230: Changed “Figure” to “Fig.”
Line 259: Changed “Figure” to “Fig.”
Line 263: Changed “Figure” to “Fig.”
Line 266: Changed “Figure” to “Fig.”
Line 286: Changed “Figure” to “Fig.”
Line 305: Changed “Figure” to “Fig.”
Line 325: Changed “Figure” to “Fig.” and “Figure 3 – Supplemental Figure 3” to “Fig. S3”
Line 327: Changed “Figure” to “Fig.”
Line 329: Changed “Figure 3 – Supplement 3” to “Fig. S4”
Line 337: Changed “Figure” to “Fig.”
Line 341: Changed “Figure” to “Fig.”
Line 345: Changed “Figure 3 – Supplement 2” to “Fig. S5”
Line 355: Changed “Figure” to “Fig.”
Line 362: Changed “ Figure 3 – Figure supplement 4” to “Fig. S6”
Line 371: Changed figure reference to specifically reference the panels that show the specific GFP fusions referenced.
Line 378: Changed “Figure 4A – 4D” to “Fig. 4A-D”

Line 380: Corrected panel reference.
Line 394: Changed “Figure 4E – 4H, Figure 4 – figure supplement 2” to “Fig. 4E-H, Fig. S7”
Line 396: Changed “Figure 4 – figure supplement 2A” to “Fig. S7A”
Line 403: Changed “Figure 4 – figure supplement 2A” to “Fig. S8A”
Line 406: Changed “Figure 4 – figure supplement 2B” to “Fig. S8B”
Line 412: Changed “Figure 4 – figure supplement 3A” to “Fig. S9A”
Line 414: Changed “Figure 4 – figure supplement 3B, 3C” to “Fig. S9B-C”
Line 419: Added a reference to supplemental figure. (5S1 or S10 depending)
Line 422: Added a reference to supplemental figure. (5S1 or S10 depending)
Line 435: Changed “Figure 4 – figure supplement 3B” to “Fig. S9B”
Line 436: Changed “Figure 4 – figure supplement 3C” to “Fig. S9C”
Line 448: Replace “knock-down” with “knockdown” for consistency.
Line 451: Replace “knock-down” with “knockdown” for consistency.
Line 452: Changed “Figure” to “Fig.”
Line 466: Added reference: “(D’Agostino et al., 2006; Jadav et al., 2008)”
Line 471: Changed “Figure” to “Fig.”
Line 471: Added reference & explanation: “where *nos-2* fails to localize to P granules (*pie-1* depletion retains *nos-2* repression in Tennehaus et al., 2001)”
Line 476: Changed “Figure 5B, 5C, Figure 5 – Figure Supplement 1” to “Fig. 5B-C, Fig. S10”
Line 479: Changed “Figure 5 – Figure Supplement 1” to “Fig. S10”
Line 481: Changed “Figure 5D, 5E” to “Fig. 5D,E”
Line 482: Changed “Figure” to “Fig.”
Line 487: Removed redundant “mRNA” in “mRNA transcripts”
Line 495: Changed “Figure 1 – figure supplement 2; Figure 2C” to “Fig. S2; Fig. 2C”
Line 502: Corrected “*gpdh-2*” to “*gpd-2*”
Line 502: Corrected capitalization of “*Dehydrogenase*”
Line 504: Removed redundant “mRNA” in “mRNA transcripts”
Line 507: Changed “Figure” to “Fig.”
Line 512: Changed “Figure” to “Fig.”
Line 518: Changed “Figure” to “Fig.”
Line 530: Changed “Figure” to “Fig.”
Line 533: Changed “Figure 6, Figure 5 – figure supplement 2” to “Fig. 6; Fig. S11”
Line 556: Replaced “stimulated” with “translationally activated” to reduce ambiguity.
Line 557: Added “shortly” to reduce ambiguity.
Line 570: Changed “Figure” to “Fig.”
Line 606-607: Added hyphens to translationally-dependent and -independent
Line 649: Added missing “an”
Line 716: Corrected capitalization of “Cal”
Line 757: Changed “for” to “compared to” to add clarity.
Line 777: Corrected link.
Line 834: Added grant reference number.

© 2020. Published by The Company of Biologists under the terms of the Creative Commons Attribution License (https://creativecommons.org/licenses/by/4.0/).

Line 874: Deleted redundant transcript.
Line 874: Deleted unnecessary “their” in “for their localization”
Line 875: Deleted “had been” from “Eight transcripts had been identified”

Line 876: Deleted “were” from “eight were P1-enriched” for clarity.
Line 876: Deleted “were” from “four were symmetrically” for clarity.
Line 877: Added “were surveyed” to make a complete sentence.
Line 890: Deleted “as they represent a subset of the mRNAs surveyed in this study” after “are shown” to reduce extraneous text in the legend.
Line 892: Deleted redundant “transcripts” from “mRNA transcripts.”
Line 895: Deleted redundant “transcripts” from “mRNA transcripts.”
Line 898: Added missing comma.
Line 904: Added caption for subfigure B to describe previously unlabeled part of figure.
Line 906: Deleted redundant “transcripts” from “mRNA transcripts.”
Line 910: Deleted redundant “transcripts” from “mRNA transcripts.”
Line 911: Deleted “and are shown” from “calculated and are shown for.”
Line 912: Deleted unnecessary “ the clustered transcripts” from “ for the clustered transcripts:”
Line 925: Replaced “and at each” with “at” to make the sentence more readable.
Line 928: Added an asterisk to “*5” to reflect what the figure shows.
Line 931: Deleted redundant “transcripts” from “mRNA transcripts.”
Line 931: Changed “P granule markers” to “P granules and P-bodies” to better reflect figure.
Line 933: Deleted unnecessary “protein” from “P granule protein marker.”
Line 933: Replaced “in concert with dual smFISH imaging of two mRNAs of either *chs-1, clu-1, cpg-2,* and the known P granule resident mRNA *nos-2*” with “and *chs-1, clu-1, cpg-2,* or *nos-2* transcripts” for brevity.
Line 934: Replaced “, DNA (DAPI, blue), and a DIC image are also shown” with “. DNA (DAPI, blue) and DIC are also shown.” for readability.
Line 936: Removed unnecessary “degree of” from “the degree of spatial overlap” for brevity.
Line 938: Removed unnecessary “in concert” from “immunofluorescence (green) in concert with smFISH”
Line 939: Removed duplicative “(magenta)”
Line 939: Replaced “a single region of co-localization” with “regions of co-localization”
Line 942-943: Replaced “sufficient to recapitulate subcellular patterns of localization.” with “sufficient for subcellular localization” for brevity.
Line 944: Replaced “The *erm-1* 3’UTR” with “the 3'UTRs of (A) erm-1, (B) imb-2, (E) cpg-2, and (G) nos-2 were” to reduce redundancy later in the legend.
Line 945: Replaced “(*mex-5p::mNeonGreen::erm-1-3´UTR*)” with “(*mex-5p::mNeonGreen::3’UTR of interest*)” to reflect changes made to line 944.
Line 946-947: Replaced “whether the 3´UTR of *erm-1* was sufficient” with “3´UTRs of interest were sufficient” to reflect changes made to line 944.
Line 947: Replaced “cell peripheral localization pattern” with "subcellular localization patterns” to reflect changes made to line 944.
Line 947-948: Replaced “*erm-1* mRNA” with “transcripts” to reflect changes made to line 944.
Line 949: Replaced “endogenous *erm-1* mRNA” with “mRNA of interest” to reflect changes made to line 944.
Line 951: Replaced “**(B)**” with “**(B and D)**”
Line 951: Replaced “Quantification of image shown in (A)” with “images shown in (A and C)”

Line 952-1044: Replaced “normalized frequency of *erm-1* mRNA and *mNeonGreen* mRNA at increasing distances from cell peripheries. **(C)** As in (A) using the *imb-2* 3´UTR sequence. **(D)** Quantification of image shown in (C) indicating the normalized frequency of *imb-2* mRNA and *mNeonGreen* mRNA at increasing distances from the nuclear periphery. **(E)** As in (A) using the *chs-1* 3´UTR sequence with channels split for the endogenous *chs-1* mRNA (left), transgenic *mNeonGreen* mRNA (middle), and the two channels merged (right). **(F)** Clustering metrics were gathered for a minimum of 5 embryos at each of five binned stages of development and the estimated mRNA content per cluster reported by the *cpg-2* probe (magenta) and *mNeonGreen* probe (green) are tabulated. **(G)** As in (E) using the *nos-2* 3´UTR sequence. **(H)** Quantification as in (F) for *nos-2* 3´UTR reporter embryos.” with “(B) *erm-1*, or (D) *imb-2* mRNA and *mNeonGreen* mRNA at increasing distances from cell peripheries. (F and H) The estimated mRNA content per

© 2020. Published by The Company of Biologists under the terms of the Creative Commons Attribution License (https://creativecommons.org/licenses/by/4.0/).

cluster from a minimum of 5 embryos at each of five binned stages of development are reported for endogenous (F) *cpg-2* or (H) *nos-2* (magenta) and *mNeonGreen* reporters (green).” for brevity.
Line 986: Changed “Figure” to “Fig.”
Line 989: Changed “Figure 1 – figure supplement 1A” to “Fig. S1A”
Line 1005: Changed “Figure” to “Fig.”
Line 1044: Deleted “individually or in combination” for brevity.
Line 1044-1045: Replaced “*nos-2* as well as three other posterior” with “*nos-2* and other posterior” for brevity.
Line 1046: Replaced “for *chs-1* mRNA (magenta) and *nos-2* mRNA (below)” with “*chs-1* mRNA (magenta, top) and *nos-2* mRNA (magenta, bottom)” to accurately represent figure.
Line 1047: Replaced “Tabulation of the impact of” with “The (C) total number of mRNA molecules and (D) average number of mRNA molecules per cluster for” to shorten legend at line 1052.
Line 1049: Changed “Figure” to “Fig.”
Line 1049: Deleted redundant “transcripts” from “mRNA transcripts.”
Line 1051-1052: Added “# indicates data analyzed in (E)” to clarify figure (D)
Line 1052: Removed “The quantifications for “total number of mRNA molecules” per embryo (C) and “average number of mRNA molecules per cluster” (D) are both shown. Depletion of MEX-3, SPN-4, or SPN-4/MEX-3 together led to increased abundance of mRNA molecules for *chs-1, cpg-2,* and *nos-2* transcripts (orange arrows). Depletion of MEX-3, SPN-4, or SPN-4/MEX- 3 in combination led to decreased clustering of *nos-2* transcripts.” For brevity
Line 1053: Fixed misspelled “conditions”
Line 1057: Replace “that prevent translation” with “that regulate translation” for accuracy.
Line 1060: Changed “Figure” to “Fig.”
Line 1137: Deleted redundant “transcripts” from “mRNA transcripts.”
Line 1139: Deleted redundant “transcripts” from “mRNA transcripts.”
Line 1159: Replaced “cluster” with “clustered” to fix tense.
Line 1170: Removed “**(A)**” because there are no subfigures.
Line 1178: Corrected “*chs-2”* to *“chs-1”*
Line 1179: Corrected “*chs-2”* to *“chs-1”*
Line 1186: Added “, left” to reflect figure.
Line 1194: Replaced “mNEONGREEN” with “mNeonGreen” to reflect proper name.
Line 1196: Removed “transcribed from cDNA” to accurately depict results.
Line 1197: Replaced *“CDS”* with *“ORF”* to accurately depict results.
Line 1197: Changed “Figure” to “Fig.”
Line 1206: Replaced “MEX-3” with *“mex-3”* to reflect proper nomenclature.
Line 1213: Changed “Figure” to “Fig.”
Line 1214: Replaced “MEX-3” with *“mex-3”* to reflect proper nomenclature.
Line 1220: Replaced “MEX-3, PIE-1, or POS-1” with “*mex-3, pie-1*, or *pos-1”* to reflect proper nomenclature.
Line 1223: Changed “Figure” to “Fig.”
Line 1224: Added “**.Quantification of mRNA clustering under RBP knockdown conditions**”, because there was no figure title.
Line 1225: Changed “Figure” to “Fig.”
Line 1273: Changed “**Figure 1 – figure supplement 1**” to “**Fig. S1**”
Line 1286: Changed “**Figure 1 – figure supplement 2**” to “**Fig. S2**”
Line 1286: Changed “**Figure 3 – figure supplement 1**” to “**Fig. S3**”
Line 1293: Changed “Figure” to “Fig.”
Line 1302: Changed “**Figure 3 - figure supplement 2**” to “**Fig. S4**” and switched Figure 3 – supplement 3 for figure 3 -figure supplement 2 to represent the actual order of presentation.
Line 1313: Changed “**Figure 3 - figure supplement 3**” to “**Fig. S5**”
Line 1318: Changed “**Figure 3 - figure supplement 4**” to “**Fig. S6**”
Line 1323: Changed “**Figure 4 - figure supplement 1**” to “**Fig. S7**”
Line 1357: Changed “**Figure 4 - figure supplement 2**” to “**Fig. S8**”
Line 1368: Changed “**Figure 4 - figure supplement 3**” to “**Fig. S9**”
Line 1384: Changed “**Figure 5 - figure supplement 1**” to “**Fig. S10**”
Line 1400: Changed “**Figure 5 - figure supplement 2.**” to “**Fig. S11**”
Line 1406: Changed “**Figure 5 - figure supplement 2**” to “**Fig. S12**”

© 2020. Published by The Company of Biologists under the terms of the Creative Commons Attribution License (https://creativecommons.org/licenses/by/4.0/).

Line 1445-1470: Changed all of the “Figure” to “Fig.”

Second decision letter

MS ID#: DEVELOP/2019/186817

MS TITLE: mRNA localization is linked to translation regulation in the *Caenorhabditis elegans* germ lineage

AUTHORS: Dylan M. Parker, Lindsay P. Winkenbach, Samuel Boyson, Matthew N. Saxton, Camryn Daidone, Zainab A. Al-Mazaydeh, Marc T. Nishimura, Florian Mueller, and Erin Osborne Nishimura

I have now received Reviewer 1's comments on your revised manuscript and have reached a decision. The reviewer's comments are appended below, or you can access them online: please go to BenchPress and click on the 'Manuscripts with Decisions' queue in the Author Area.

The overall evaluation is positive, and I would like to publish your study in Development, after you have incorporated the text changes suggested by Reviewer 1. Please submit a revised manuscript with the changes highlighted and detailed in a point-by-point response.

Reviewer 1

Advance summary and potential significance to field

Patterns of subcellular mRNA distribution are both striking and intriguing, but very few studies have looked into what drives these patterns and whether they are required for proper development. This investigation reveals several mRNAs that exhibit membrane and RNP localization through diverse regulatory mechanisms.

Comments for the author

In this revision, the authors have addressed most of the reviewer comments. Some concerns about figure 7 need to be address (below). Parts of the abstract and introduction are difficult to read, so I’ve included some suggested edits that may improve clarity.

Abstract

- Sentence 1. Add comma between transcription and thereby
- Sentence 2. Replace “In studying this patterning, we discovered” with “We observed”
- Sentence 3. Insert “The subcellular distribution of” before transcripts
- Sentence 5. Consider “By uncoupling translation from mRNA localization, we untangled a long-standing question: Are mRNAs directed to P granules to be translationally repressed, or do they accumulate there as a consequence of this repression?
- Sentence 6. We found that translational…
- Last sentence. Consider replacing with “These results implicate translational repression as a means to package and deliver essential maternal transcripts to the progenitor germ lineage for later translation.”

Introduction

- Edit paragraph one to read:
    - The progression of life from two gametes to an embryo involves the transfer of gene expression responsibilities from the parental to zygotic genomes. In animals, this maternal-to-zygotic transition requires a pause in transcription during late oogenesis, fertilization, and the first stages of zygotic development (Hamm and Harrison, 2018; Robertson and Lin, 2015; Schulz and Harrison, 2019; Vastenhouw et al., 2019). Until zygotic transcription resumes, cell-type transcriptome differences

© 2020. Published by The Company of Biologists under the terms of the Creative Commons Attribution License (https://creativecommons.org/licenses/by/4.0/).

in the early embryo arise through post-transcriptional mechanisms acting on mRNAs inherited from the parental gametes.

- Sentence two of paragraph five, consider:
  - Therefore, maternal asymmetric mRNAs seem to be necessary for cellular diversification during early development. In this study, we explore the mechanisms and functions of this patterning.
- Paragraph seven, consider:
  - Understanding the functional roles of P granules (and other phase-separated condensates) is a current major challenge. In early embryos, P granules are dispersed in the cytoplasm and highly dynamic (Hird et al., 1996; Strome and Wood, 1982), but later grow into larger granules that coalesce around the nucleus (Sheth et al., 2010). Here they extend the nuclear pore complex environment and branch into more specialized condensates such as mutator foci (Phillips et al., 2012) and Z-granules (Wan et al., 2018). Worms can recover from P granule disruption in early embryonic stages to properly specify the germline (Gallo et al., 2010), but later or sustained dysregulation leads to perturbations in germ cell development (Wang et al., 2014), disruption of gene expression regulatory control (Campbell and Updike, 2015; Updike et al., 2014; Voronina et al., 2012) and fertility defects (Kawasaki et al., 2004; Spike et al., 2014; Wang et al., 2014). The reasons why mRNAs associate with P granules may depend on the individual transcript or developmental stage, but functions such as translational repression, RNA processing, small RNA-based regulation, or piRNA licensing are possibilities, based on P granule composition.
- Split sentence two of paragraph eight to read:
  - Indeed, the well-studied, P granule-resident mRNA nos-2 is also translationally repressed at early embryonic stages. Later, this repression is relieved when NOS-2 becomes essential for germline development (D'Agostino et al., 2006; Jadhav et al., 2008; Subramaniam and Seydoux, 1999).
- Last paragraph of introduction. As I noted in my initial review, I still think that you'll run into trouble arguing that there is a general difference between the subcellular dispersal of maternal and zygotic transcripts, given the constrain on cell size after zygotic transcription kicks in. Therefore, I'd suggest replacing the middle sentence of this last paragraph with:
  - Our findings also suggest that the subcellular patterning of maternally inherited transcripts is a common feature of early embryogenesis.
- "relieved" is misspelled before the D'Agostino and Jadhav references in the large paragraph before "Quantification strategies to characterize mRNA patterning"
- Third sentence under the paragraph headed by "Clustered transcripts colocalize…," I don't think most would agree that P granules are called something different later in development. Consider:
  - "In *C. elegans*, germ granules are specifically called P granules (Fig. 3A) (Seydoux 2018; Marnik and Updike 2019), and they segregate to the P lineage with each successive cell division."
- "Given that we observed *chs-1*, *clu-1*, and *cpg-2* mRNAs clustered and progressing down the P lineage, we hypothesized that they might be within P granules."
- Paragraph one following "3'UTRs were sufficient…,"second sentence. It is unclear whether these nNG reporters are single copy integrations via CRISPR or MosSci, or whether they are expressed from multi-copy arrays. Please clarify. If expressed from arrays, in the second paragraph under this heading, I'd recommend removing the comparison of imb-2 mRNA quantity and what it might suggest about mRNA stability. It could just be that one transgene is expressed better than the other. If single copy integrations, then a comparison can be made. Then the same goes for the chs-1 transcript destabilization statement in the next paragraph.
- Figure 7. In general, colocalization in this figure is very hard to see, and doesn't appear to be quantified. Can the figures be enlarged with improved resolution, and can colocalization data accompany this figure?
- Second to the last paragraph of the discussion. While not necessarily needed for this paper, the model that transcripts are directed to membranes as they are passably dragged behind the growing peptide could be tested by removing the signal peptide and seeing whether transcripts are still directed to these areas. Is there data to support this? Consider making

© 2020. Published by The Company of Biologists under the terms of the Creative Commons Attribution License (https://creativecommons.org/licenses/by/4.0/).

it a brief point of discussion.

Second revision

Author response to reviewers' comments

Dear Reviewers,

Thank you for your circumspect review of our manuscript. The textual recommendations were very thoughtful and greatly improved our manuscript. We appreciate the time and effort it took to make these recommendations.

We have also added computational analysis to Figure 7 (heat shock induced translational disruption) in a new Figure 7B that illustrates the increasing percentage of RNA clusters that localize to P granules upon heat shock. In addition, we added Figure S13A that illustrates computationally the effect of heat shock on inducing RNA clusters and increasing their size and Figure S13B that illustrates the raw number of RNA clusters that overlap with P granules. The addition of this computational analysis has strengthened the manuscript.

With the additional textual changes, the manuscript is still 150 or so words above the 7000 word limit. Please instruct us as to whether you would like to see us edit the work down below the limit or whether there is some leniency there.

Thank you again for your attention to detail and thoughtful review.

Point-By-Point Response

Abstract

- Sentence 1. Add comma between transcription and thereby
  - **done**
- Sentence 2. Replace “In studying this patterning, we discovered” with “We observed”
  - **done**
- Sentence 3. Insert “The subcellular distribution of” before transcripts
  - **done**
- Sentence 5. Consider “By uncoupling translation from mRNA localization, we untangled a long- standing question: Are mRNAs directed to P granules to be translationally repressed, or do they accumulate there as a consequence of this repression?
  - **done**
- Sentence 6. We found that translational…
  - **done**
- Last sentence. Consider replacing with “These results implicate translational repression as a means to package and deliver essential maternal transcripts to the progenitor germ lineage for later translation.”
  - **done**

Introduction

- Edit paragraph one to read:
  - The progression of life from two gametes to an embryo involves the transfer of gene expression responsibilities from the parental to zygotic genomes. In animals, this maternal-to-zygotic transition requires a pause in transcription during late oogenesis, fertilization, and the first stages of zygotic

© 2020. Published by The Company of Biologists under the terms of the Creative Commons Attribution License (https://creativecommons.org/licenses/by/4.0/).

development (Hamm and Harrison, 2018; Robertson and Lin, 2015; Schulz and Harrison, 2019; Vastenhouw et al., 2019). Until zygotic transcription resumes, cell-type transcriptome differences in the early embryo arise through post-transcriptional mechanisms acting on mRNAs inherited from the parental gametes.

- **done**

- Sentence two of paragraph five, consider:
  - Therefore, maternal asymmetric mRNAs seem to be necessary for cellular diversification during early development. In this study, we explore the mechanisms and functions of this patterning.
    - **done**
- Paragraph seven, consider:
  - Understanding the functional roles of P granules (and other phase-separated condensates) is a current major challenge. In early embryos, P granules are dispersed in the cytoplasm and highly dynamic (Hird et al., 1996; Strome and Wood, 1982), but later grow into larger granules that coalesce around the nucleus (Sheth et al., 2010). Here they extend the nuclear pore complex environment and branch into more specialized condensates such as mutator foci (Phillips et al., 2012) and Z-granules (Wan et al., 2018). Worms can recover from P granule disruption in early embryonic stages to properly specify the germline (Gallo et al., 2010), but later or sustained dysregulation leads to perturbations in germ cell development (Wang et al., 2014), disruption of gene expression regulatory control (Campbell and Updike, 2015; Updike et al., 2014; Voronina et al., 2012) and fertility defects (Kawasaki et al., 2004; Spike et al., 2014; Wang et al., 2014). The reasons why mRNAs associate with P granules may depend on the individual transcript or developmental stage, but functions such as translational repression, RNA processing, small RNA-based regulation, or piRNA licensing are possibilities, based on P granule composition.
    - **done**
- Split sentence two of paragraph eight to read:
  - Indeed, the well-studied, P granule-resident mRNA nos-2 is also translationally repressed at early embryonic stages. Later, this repression is relieved when NOS-2 becomes essential for germline development (D'Agostino et al., 2006; Jadhav et al., 2008; Subramaniam and Seydoux, 1999).
    - **done**
- Last paragraph of introduction. As I noted in my initial review, I still think that you'll run into trouble arguing that there is a general difference between the subcellular dispersal of maternal and zygotic transcripts, given the constrain on cell size after zygotic transcription kicks in. Therefore, I'd suggest replacing the middle sentence of this last paragraph with:
  - Our findings also suggest that the subcellular patterning of maternally inherited transcripts is a common feature of early embryogenesis.
    - **We amended our language on Page 6 to reflect concerns about our ability to call zygotically expressed transcripts as patterned given their smaller size.**
    - **done**

Results:

- "relieved" is misspelled before the D'Agostino and Jadhav references in the large paragraph before "Quantification strategies to characterize mRNA patterning"
  - **fixed**
- Third sentence under the paragraph headed by "Clustered transcripts colocalize…," I don't think most would agree that P granules are called something different later in development. Consider:
  - "In C. elegans, germ granules are specifically called P granules (Fig. 3A) (Seydoux 2018; Marnik and Updike 2019), and they segregate to the P lineage with each successive cell division."

© 2020. Published by The Company of Biologists under the terms of the Creative Commons Attribution License (https://creativecommons.org/licenses/by/4.0/).

  - **Good point. We fixed this.**
- "Given that we observed chs-1, clu-1, and cpg-2 mRNAs clustered and progressing down the P lineage, we hypothesized that they *might* be within P granules."
  - **fixed**
- Paragraph one following "3'UTRs were sufficient…,"second sentence. It is unclear whether these nNG reporters are single copy integrations via CRISPR or MosSci, or whether they are expressed from multi-copy arrays. Please clarify. If expressed from arrays, in the second paragraph under this heading, I'd recommend removing the comparison of imb-2 mRNA quantity and what it might suggest about mRNA stability. It could just be that one transgene is expressed better than the other. If single copy integrations, then a comparison can be made. Then the same goes for the chs-1 transcript destabilization statement in the next paragraph.
  - **These are single copy insertions using Cas9 mediated insertions into the MOSCI integration sites. We have amended the text to reflect this key point. Thank you.**

Figure 7:
- Figure 7. In general, colocalization in this figure is very hard to see, and doesn't appear to be quantified. Can the figures be enlarged with improved resolution, and can colocalization data accompany this figure?
  - **We have enlarged the figure.**
  - **We have quantified the colocalization of these images and included quantification (Figure 7B and Supplemental Figure S13). A new figure 7B legend is also included.**

Discussion:
- Second to the last paragraph of the discussion. While not necessarily needed for this paper, the model that transcripts are directed to membranes as they are passably dragged behind the growing peptide could be tested by removing the signal peptide and seeing whether transcripts are still directed to these areas. Is there data to support this? Consider making it a brief point of discussion.
  - **There are no discernable signal peptides in any of our membrane-localized proteins. Instead, we hypothesize that the membrane associated domains of these proteins may fulfill this role by directing localization. We have amended the wording in the discussion to reflect this.**

In addition, we have added:
- **New computational analysis of Figure 7 as Figure 7B**
  - **New Figure 7B figure legend**
  - **New source data for Figure 7B**
- **New computational analysis supporting images of Figure 7 that are now Figure S13A and S13B**
  - **Figure legends on the same page.**
  - **New source data for Figure S13A**

Third decision letter

MS ID#: DEVELOP/2019/186817

MS TITLE: mRNA localization is linked to translation regulation in the *Caenorhabditis elegans* germ lineage

AUTHORS: Dylan M. Parker, Lindsay P. Winkenbach, Samuel Boyson, Matthew N. Saxton, Camryn Daidone, Zainab A. Al-Mazaydeh, Marc T. Nishimura, Florian Mueller, and Erin Osborne Nishimura
ARTICLE TYPE: Research Article

© 2020. Published by The Company of Biologists under the terms of the Creative Commons Attribution License (https://creativecommons.org/licenses/by/4.0/).

I am happy to tell you that your manuscript has been accepted for publication in Development, pending our standard ethics checks. Either now or during the page proof stage, you should change "transcriptional" to "translational" on line 46 of your Abstract. I am OK with your article exceeding the word limit by a bit. The journal may ask you to trim it.

© 2020. Published by The Company of Biologists under the terms of the Creative Commons Attribution License (https://creativecommons.org/licenses/by/4.0/).
